# Nongeminate Radiative Recombination and *V*_oc_ in Organic Solar Cells Enhanced by a Charge Transporter/Absorber Interface Change

**DOI:** 10.1021/acsaem.5c02775

**Published:** 2025-12-03

**Authors:** Francisco Bernal-Texca, Chiara Cortese, Mariia Kramarenko, Jordi Martorell

**Affiliations:** † ICFO – Institut de Ciencies Fotoniques, The Barcelona Institute of Science and Technology, 08860 Castelldefels, Barcelona, Spain; ‡ Departament de Física, Universitat Politécncnica de Catalunya, 08222 Terrasa, Spain

**Keywords:** organic solar cells, nongeminate recombination, open-circuit voltage, fluorescence quantum yield, electron transport layer, interfacial traps

## Abstract

The fluorescence quantum yield (FQY) defines the ultimate open-circuit voltage (*V*_oc_) and efficiency limit in single-junction solar cells. However, enhancing solar cell efficiency by increasing the fluorescence is rarely approached provided in the majority of solar cells, the *V*_oc_ is assumed to be essentially limited by a large fraction of nonradiative recombination when compared to the radiative one. Here, we show that in completed PM6:Y6 organic solar cells (OSCs), the FQY is 1 order of magnitude higher than in the blend alone. Such an FQY is further enhanced by 18% through the incorporation of an ultrathin LiF layer between the blend and the ZnO electron transport layer (ETL), which reduces interfacial oxygen molecules acting as electron traps. The FQY enhancement leads to an increase in quasi-Fermi level splitting (QFLS), resulting in a *V*_oc_ gain which we measured to be as large as 12 mV. Using the two-diode model, we confirm that this pathway for enhancing QFLS has the potential to be applicable to other single-junction solar cells where radiative recombination is present at the interface between charge selective contacts and the absorber material.

## Graphical abstract

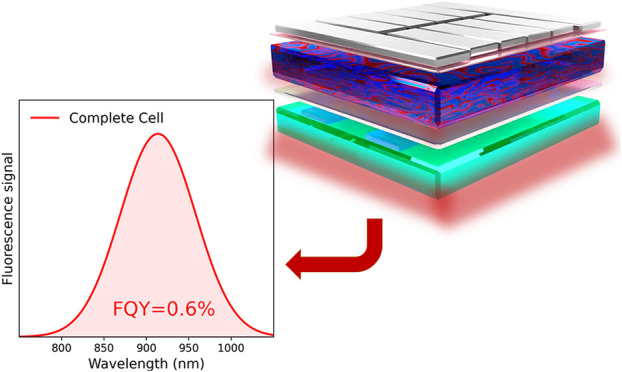

## 1 Introduction

One of the severest limitations to high conversion of sunlight to electrical energy when using OSCs is the large open-circuit voltage (*V*_oc_) loss, which roughly doubles the one from perovskite or silicon cells. Such loss is mostly the result of two main factors: a large fraction of nonradiative recombination and the driving force needed to achieve a spatial separation for the electron and hole pair from the excitons or charge transfer (CT) states created after photon absorption.^1–3^

Most of the strategies implemented to date to reduce such voltage loss have considered a material modification approach. Recently, the synthesis of new materials was considered to obtain organic blends that required a small driving force for charge separation.^4–7^ In a different approach, it was shown that an increase in the organic active layer crystallinity, leading to CT state delocalization would eventually result in the mitigation of the CT state deactivation.^8,9^ A reduced nonradiative loss can also be achieved by a molecular packing leading to delocalization of the electron wave function.^10,11^

A radically different path to reduce *V*_oc_ losses, applicable to any kind of planar geometry solar cell, organic or inorganic, can be rooted to increasing the percentage contribution of fluorescence among the different recombination channels,^12^ to subsequently implement an angular restriction of the fluorescence emission cone.^13^ Although when following such approach, the gain in *V*_oc_ can be extraordinary, it has not been implemented yet, mostly because of the relatively low external FQY of the majority of solar cell materials, where nonradiative recombination dominates over recombination via photon emission.^14,15^ Recent studies on completed OSCs have demonstrated that once charge separation is reached and a QFLS is established, the dark current characteristics of the *J*–*V* curve can be better described by the two-diode model, which considers separately the contribution to recombination from midgap trap states and direct band-to-band transitions.^16,17^ If the latter were to be dominated by fluorescence, this in itself would imply a gain in *V*_oc_, which could be further increased by applying an angular restriction for photon emission. The combination of both would result in a path to dramatically reduce *V*_oc_ losses. In the current work, we demonstrate that fluorescence in complete OSCs is increased by one order of magnitude when compared to the fluorescence of the active layer blend alone. We also demonstrate that by reducing the charge trapping states at the interface between the ETL and the organic blend, the fluorescence is enhanced, resulting in a significant increase in *V*_oc_.

## 2 Experimental Methods

### 2.1 Materials and Solution Preparation

The polymer PM6 (Batch No. YY22230CH100, purchased from 1-material) and Y6 (Batch No. DW9132P, purchased from 1-material) were used as received without further purification. Ag pellets from Kurt J. Lesker Company (≥99.99% purity, 1/8″ diameter × 1/8″ long), MoO_3_ from Alfa Aesar (≥99.95% purity), and powder LiF (<100 μm, ≥99.98% metal trace basis) from Sigma-Aldrich were used as purchased. 0.15 M sol–gel ZnO (SG-ZnO) precursor solutions were prepared by dissolving zinc acetate dihydrate (Sigma-Aldrich, 263 mg) and ethanolamine (Sigma-Aldrich, 73 μL) in 2-methoxyethanol (Sigma-Aldrich, 8 mL), and the solution was then stirred at 60 °C for 2 h at 3000 rpm in air and then kept stirring at room temperature overnight. Solutions of PM6:Y6 (1.0:1.2 by weight) with a total concentration of 16 mg mL^–1^ were prepared in chloroform (Sigma-Aldrich, ≥99.0%) and stirred overnight at 200 rpm. Additive 1-chloronaphthalene (Sigma-Aldrich, 0.5% by volume) was added to the PM6:Y6 blend 1 h before the spin-coating, with the blend kept at a temperature of 40 °C.

### 2.2 Solar Cell Fabrication

First, precleaned ITO-patterned glass substrates (LumTec) were treated for 10 min in UVO. SG-ZnO precursor solution was spin-coated with parameters optimized for a layer thickness of ≈10 nm (5000 rpm for 30 s). After thermally annealing at 150 °C in air (relative humidity <40%) for 30 min, the ZnO-coated samples were transferred into a nitrogen-filled glovebox. For ZnO/LiF cells, 5 nm of LiF was thermally evaporated at a rate of 0.6 Å s^–1^ prior to the active layer spin-coating. PM6:Y6 blend with a controlled thickness of ≈100 nm was spin-coated in a nitrogen-filled glovebox. The hole transport layer (MoO_3_, 4 nm, rate 0.6 Å s^–1^) and the top electrical contact (Ag, 100 nm, rate 1 Å s^–1^) were deposited sequentially by thermal evaporation (chamber pressure <5 × 10^–6^ mbar). A shadow mask was used to define the cell area as the overlap between ITO and top Ag electrodes, for a total of 8 cells per sample, with an active layer area of 0.06 cm^2^.

### 2.3 Characterization

The *J*–*V* curves of all devices were measured with a Keithley 2420 source meter under 1 sun, AM1.5G spectrum from a solar simulator (ABET Sol3A). The illumination intensity of the lamp (xenon lamp, 300 W, USHIO) was calibrated using a Hamamatsu silicon photodiode, certified by ISE Fraunhofer. Spectrally resolved EQEs were measured using benchtop equipment (QEX10, PV Measurements, Inc.) at 130 Hz, using the spectral response of a calibrated silicon cell as a reference. Light transmission measurements were acquired using a Lambda 1050 UV/VIS/NIR spectrometer from Perkin Elmer. The XPS/UPS measurements were performed with a SPECS PHOIBOS 150 hemispherical analyzer under ultrahigh-vacuum conditions (10^‑10^ mbar) at the Institut Català de Nanociència i Nanotecnologia.

## 3 Results and Discussion

### 3.1 Photovoltaic Performance

We consider an organic solar cell of the PM6:Y6 blend in an inverted architecture: Glass/ITO/ETL/PM6:Y6/MoO_3_/Ag, graphically displayed in Figure 1a, where the ETL is composed of a single ZnO layer in the reference device or, in a second cell, of an ultrathin LiF evaporated layer of 5 nm on top of the 10 nm ZnO layer.

**Figure 1:**
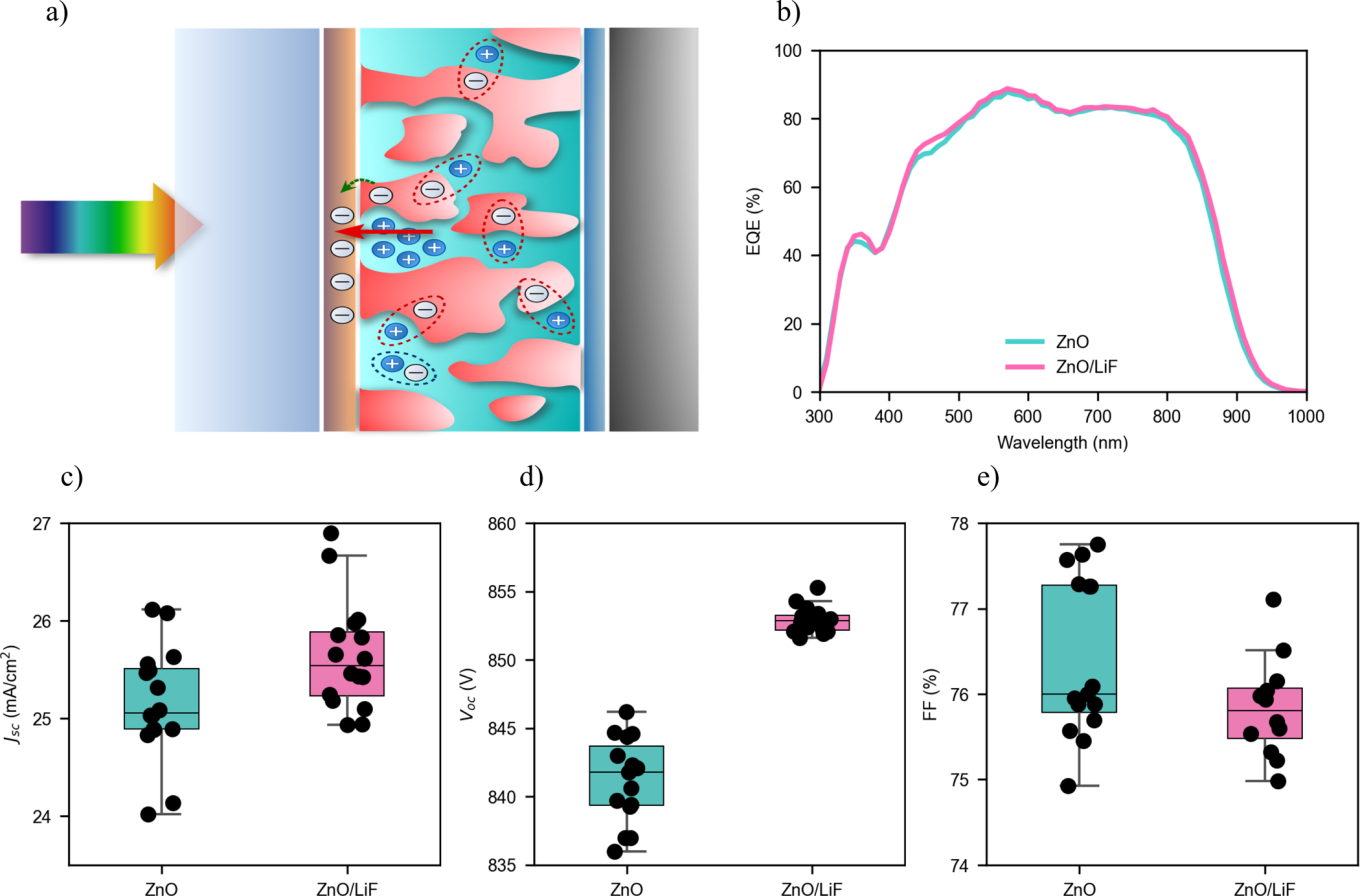
Device structure and performance. (a) Schematic illustration of a PM6:Y6 blend solar cell where the red arrow indicates the buildup of an electric field at the interface separating the ETL or HTL from the blend. CT states are indicated with a red dotted line, while excitons are indicated with a blue dotted line. (b) External quantum efficiency (EQE) of the PM6:Y6 cells with ETL being ZnO and ZnO/LiF. (c–e) Statistics of the *J*_sc_, *V*_oc_, and FF, respectively, from the completed cells measured under 1 sun AM1.5G illumination when ETL is a ZnO layer (left) and a ZnO layer with a LiF layer deposited on top (right). Statistics on the power conversion efficiency (PCE) can be seen in Figure S8.

We first evaluate the effect on the photovoltaic performance change from such cells when the interface between ZnO and the blend is modified by the addition of the LiF layer. From the external quantum efficiency (EQE) plot displayed in Figure 1b and the box plot of Figure 1c, it can be seen that *J*_sc_ slightly increases with the addition of the LiF layer. The EQE has a slight increase in the 400–600 nm region, which can be attributed to the enhanced transmission in that region (Figure S1). The standard box plots from Figure 1d show a 12 mV *V*_oc_ increase when the LiF is added on top of ZnO. Additional experiments in Figure S2 demonstrate that there is always a consistent increase in *V*_oc_ that ranges from 4 to 12 mV when the LiF layer is added. Fill factor (FF) slightly decreases with the addition of LiF, which can be attributed to an increase in the series resistance, as evidenced in Figure S3 from the *I*–*V* curves of the half-device ITO/ETL/Ag and in Figure S22 from the dark *J*–*V* curve analysis of the full devices. The largest efficiency of 17.28% corresponds to the device containing LiF, as can be seen in Table 1 of the summarized photovoltaic parameters. Note that considering that FF decreases and *J*_sc_ slightly increases with the addition of the LiF, the main parameter contributing to the PCE enhancement is the *V*_oc_. Further testing showed that LiF passivation maintains its benefits across different organic active layer thicknesses (Figure S4). Moreover, we evaluated the effect of the LiF addition across two different organic active layer blends (Figure S5) and observed a performance increase in both cases. The LiF passivation also demonstrated a similar positive effect even in devices fabricated with commercially available ZnO nanoparticle ink (Figure S6), which underscores the general applicability of the approach. To assess the stability of the ZnO/LiF passivated devices, Figure S7 shows the recorded performance under continuous light illumination, demonstrating enhanced stability compared to the ZnO case.

**Table 1:** Photovoltaic Parameters of the Different Devices Measured at 1 sun AM1.5G Conditions^a^

cell type	*J*_sc_ ^b^ [mA/cm^2^]	*V*_oc_ [mV]	FF [%]	PCE (PCE_max_) [%]
ZnO	25.17 ± 0.6	841.2 ± 3.1	76.41 ± 0.93	16.18 ± 0.49 (17.02)
ZnO/LiF	25.79 ± 0.6	852.9 ± 0.9	75.83 ± 0.58	16.54 ± 0.36 (17.28)

a The averaged photovoltaic parameters of each cell type are obtained from 14 devices. Experimental *J*–*V* curves can be found in Figure S9.

b The *J*_sc_ was obtained by integrating the EQE spectra.

### 3.2 Trapping States Linked to Oxygen Vacancies

The chemical transformation of the ETL/PM6:Y6 interface when the LiF is deposited between such layers can be monitored by high-resolution X-ray photoelectron spectroscopy (XPS) of the O 1s spectra. It is generally accepted that the surface of a ZnO film contains many oxygen vacancies (Ovs) and adsorbed oxygen molecules on the surface, which may act as electron traps for the free electrons that diffuse from the LUMO of the Y6 to the ZnO conduction band once the CT states separate into free electrons and holes,^18–20^ as shown schematically in Figure 1a. In Figure 2a, one sees that the reference sample XPS spectrum can be deconvoluted into three peaks which reveal the presence of three distinct binding states attributed to oxygen bound to the lattice (Zn–O, 531.04 eV), Ovs (532.12 eV), and hydroxyl groups (H–O, 532.85 eV), the latter ones likely originating from adsorbed water or residuals from the alcohol solvent used in the sol–gel reaction when depositing the ZnO layer.^21,22^ As seen in Figure 2b, when the LiF layer is added on top of the ZnO, the XPS O 1s spectrum exhibits a new peak attributed to Li–O (533.28 eV), which dominates over the other two peaks, with the contribution of the Ovs limited to 14.18% of the total. The formation of the Li–O would imply a reduction of the adsorbed oxygen molecules on the surface and, consequently, a reduction of the free electron capturing capacity by traps at the interface.^23–25^ To further explore the passivation role of the LiF in the ZnO surface, we employed ultraviolet photoelectron spectroscopy (UPS) to determine the Fermi energy level and valence band of both ZnO and ZnO/LiF. The UPS spectra in Figure 2c show a decrease in the work function (*W*_f_) of the ZnO with the addition of the LiF, from 3.62 to 3.12 eV. This reduction explicitly confirms the passivation effect of the LiF layer on the ZnO surface. Additionally, Section S.1 presents an analysis of the band bending at the interface where the energy offset of the electron transfer is reduced with the addition of the LiF.

**Figure 2:**
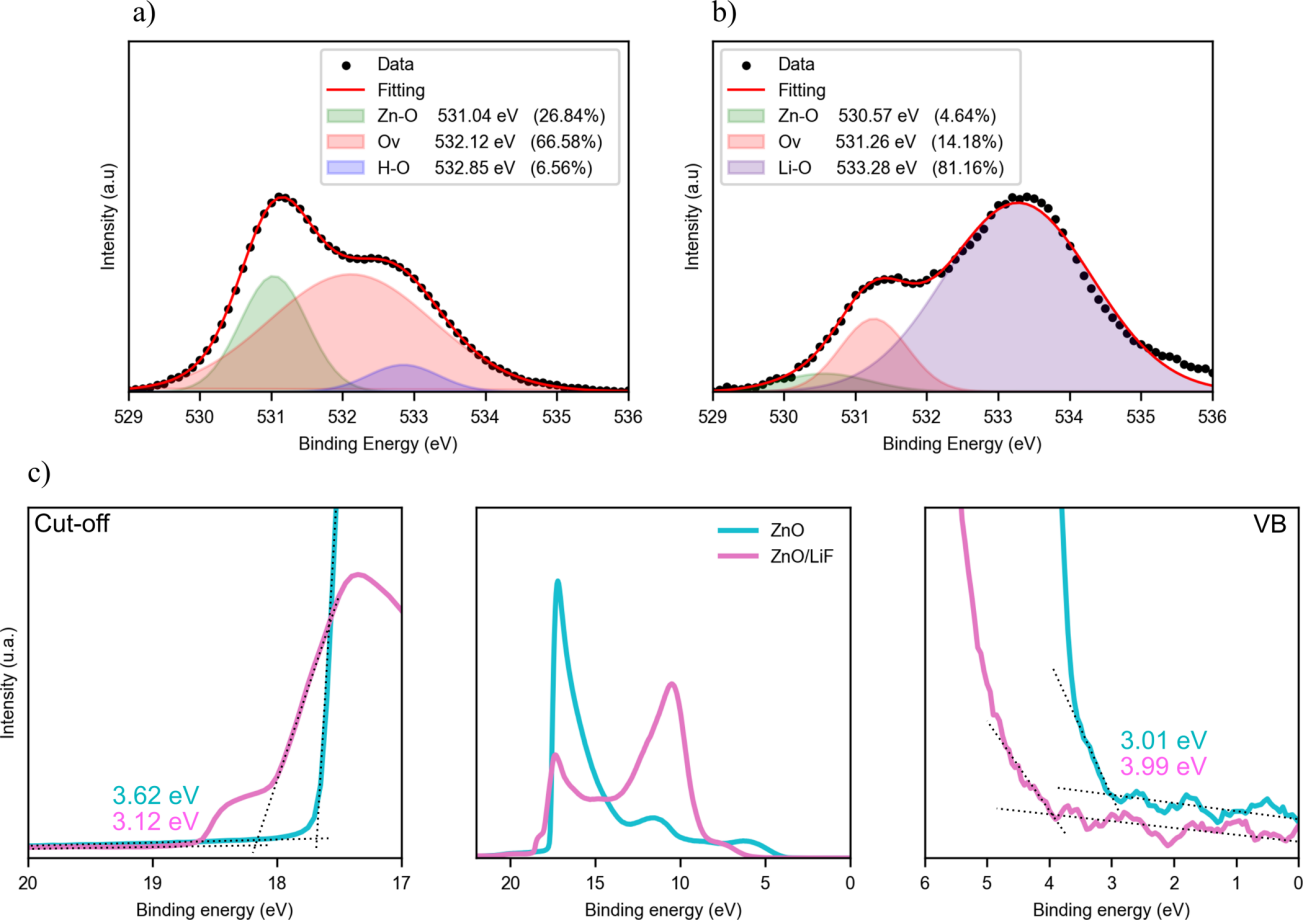
Surface analysis. (a) O 1s X-ray photoelectron spectroscopy (XPS) experimental measurement (black dots) and fitting (red line) for a ZnO layer deposited on top of an ITO substrate. (b) The same as in (a) when a LiF layer is deposited on top of the ZnO layer. The percentage values of the area of each deconvoluted peak relative to the fitted red curve are given in the legend. (c) UPS spectra of ZnO and ZnO/LiF on glass/ITO substrates. The dashed lines on the left and right panels of the UPS spectra mark the secondary electron cutoff and valence band edge, respectively.

The change in the ZnO surface composition and the increase in *V*_oc_ brought by the deposition of the LiF layer suggest that there are, at least, two competing recombination mechanisms near the ETL/active layer interface that govern the *V*_oc_ dynamics. According to the work reported in ref 16, such two competing mechanisms, once the QFLS is established, are a recombination via midgap trap states and a direct recombination composed of a radiative and nonradiative component.

### 3.3 Steady-State Fluorescence

To explore the impact of the passivation of the trapping states and establish the origin and relative contribution of the direct band-to-band recombination in governing *V*_oc_, below we consider steady-state and time-resolved fluorescence measurements on the completed cells. Details of the experimental setup to measure the FQY in steady state are provided in Section S.2. When following the procedure described in Section S.3, we estimated such an FQY to be 0.52%. To elucidate the origin of such fluorescence, we first measured the acceptor exciton emission from a 100 nm Y6 film deposited on glass as shown in Figure 3a. Such emission is centered at 930 nm, in agreement with previously reported measurements.^26,27^ We then considered the fluorescence from a 100 nm PM6:Y6 blend layer on the glass. As shown in Figure 3a, when the PM6:Y6 blend is formed, the Y6 fluorescence is largely quenched, likely from the formation of the CT states, which are known to deactivate nonradiatively.^28^ Interestingly, fluorescence is recovered when the complete solar cell is considered, as illustrated in Figure 3b. Although the peak of the emitted fluorescence photons from the complete solar cells is near the Y6 exciton fluorescence peak, such former fluorescence exhibits a close to a Gaussian profile distinct from the spectral profile of the Y6 molecule alone, suggesting that, to a large extent, it cannot be attributed solely to the Y6 exciton reconstruction and subsequent radiative electron–hole pair deactivation. The emitted signal from the complete cells peaks at 920 nm. When converted to photon energy, it would roughly match an energy that may correspond to QFLS, as schematically shown in the energy level diagram in Figure 3c. When a LiF layer is deposited on top of the ZnO layer, as shown in Figure 3b, the total photon emission intensity of complete cells increases by 18%, resulting in an FQY of 0.6%. This would suggest that when considering recombination, the relative percentage of radiative recombination increases because the number of defects is reduced. Adding LiF reduces the amount of absorbed oxygen molecules which act as electron traps at the interface.

**Figure 3:**
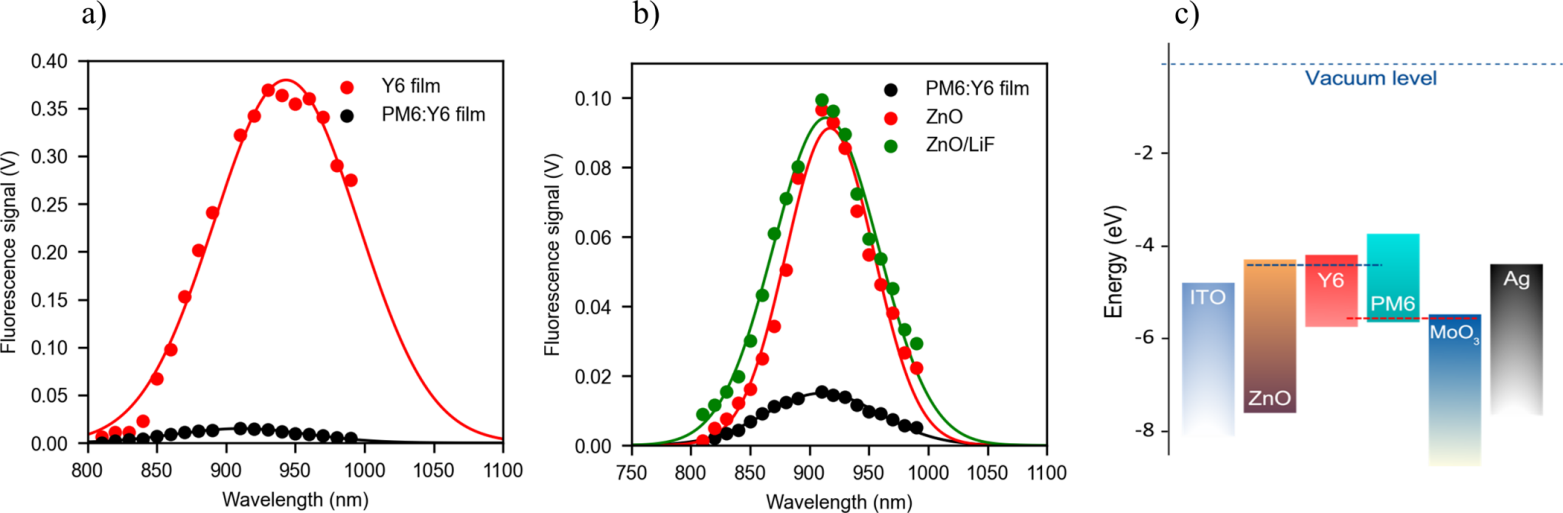
Steady-state fluorescence. (a) Fluorescence spectra of the Y6 (red solid dots) and PM6:Y6 (black solid dots) thin films deposited on glass. (b) Fluorescence spectra of the complete PM6:Y6 cells with ETL being ZnO (red solid dots) and ZnO/LiF (green solid dots). The fluorescence signals were filtered using a total of nineteen 10 nm bandpass filters with peaked transmission wavelengths ranging from 810 to 990 nm. The experimental data points shown are corrected by the corresponding blend absorptions at each wavelength. The solid lines correspond to Gaussian fits of the corrected experimental data points. The fluorescence spectrum of the PM6:Y6 film deposited on glass (black solid dots) is included for comparison. (c) Energy diagram of the PM6:Y6 solar cell. The HOMO and LUMO values of the different layers were obtained from ref 32.

We can use the two-diode model to determine the recombination current density change that has the largest impact on the increase in the FQY measured. In such a model, there are essentially three recombination channels that govern the *V*_oc_ dynamics. A current density is associated with recombination via trap states (*J*_t_), and a current density is associated with direct band-to-band recombination (*J*_d_), which is divided between nonradiative (*J*_nr_) and a radiative (*J*_r_) parts.^29^ The *J*_t_ and *J*_d_ at 0 V can be estimated from the *V*_oc_ versus light intensity shown in Figure S20 and from applying the two-diode model as explained in Section S.5 of the Supporting Information. We may assume that when the LiF is added, *J*_r_ remains largely unchanged, provided it is essentially determined by dipole transition strength and the cell architecture that determines the electromagnetic environment for such a dipole to radiate. A very thin LiF layer may cause a negligible change to such a dipole or its electromagnetic environment, as derived by optical simulations in Section S.4 and reported in Table 2. Then, as explained in detail in Section S.5, using the two-diode model, we determine the (*J*_t_, *J*_nr_) pairs that would agree with the 18% FQY increase observed. Such an FQY increase results in a *V*_oc_ increase of 4 mV when the FQY for the reference cell is 0.52% as seen in Figure S23. Note that such an FQY corresponds to an average, and if the FQY for the reference cell is as high as 0.81%, the two-diode model predicts an increase in *V*_oc_ of 12 mV for the same 18% FQY increase. The two-diode model voltage increase predictions based on the FQY nominal value and its changes are in very good agreement with the experimentally measured voltage increases which range from 4 to 12 mV. A summary of the values obtained using the two-diode model for the 4 and 12 mV increases is found in Table 2. Our approach also provides that the total nonradiative energy loss on the *V*_oc_ goes from 0.240 to 0.236 eV, on average. Those values are comparable with those reported in the literature, obtained from electroluminescence experiments.^30,31^ Further details of the model can be found in Sections S.4, S.5, and S.6.

**Table 2:** Comparison between Experimental *V*_oc_ and Calculated *V*_oc_ Using the Two-Diode Model from the *J*–*V* Curve under Illumination for a ZnO Cell and Two ZnO/LiF Cells Displaying Different *V*_oc_ Gains^a^

device	exp. *V*_oc_ [mV]	fit *V*_oc_ [mV]	*J*_t_ [A/cm^2^]	*J*_r_ [A/cm^2^]	*R*_s_ [Ω·cm^2^]	calculated FQY [%]
ZnO	840.3	840.2	0.89 × 10^–10^	1.73 × 10^–20^	1.4	0.50
ZnO/LiF	845.4	844.2	0.78 × 10^–10^	1.74 × 10^–20^	3.2	0.59
ZnO/LiF	849.3	848.2	0.42 × 10^–10^	1.74 × 10^–20^	3.8	0.68

a The most relevant parameters from the model are reported: radiative direct recombination current density (*J*_r_), trap recombination current density (*J*_t_), and series resistance *R*_s_, as well as the FQY calculated with the model.

### 3.4 External Fluorescence Governing the *V*_oc_ Dynamics

To study the *V*_oc_ dynamics, we pumped the finished LiF cell in open-circuit conditions with an ∼0.38 μJ/cm^2^ 120 fs laser pulse tuned at 590 nm, which excites preferentially the PM6 polymer over the Y6 molecule, which may lead to energy transfer from the polymer to the molecule. We measured the emitted light from the completed cell using a photomultiplier tube (PMT), as shown schematically in Figure 4a. As seen in Figure 4b, the emitted light signal, which is proportional to the number of fluorescence-emitted photons, peaks approximately at 920 nm, roughly at the same wavelength that the steady-state fluorescence is at maximum when we consider the complete cell.

**Figure 4:**
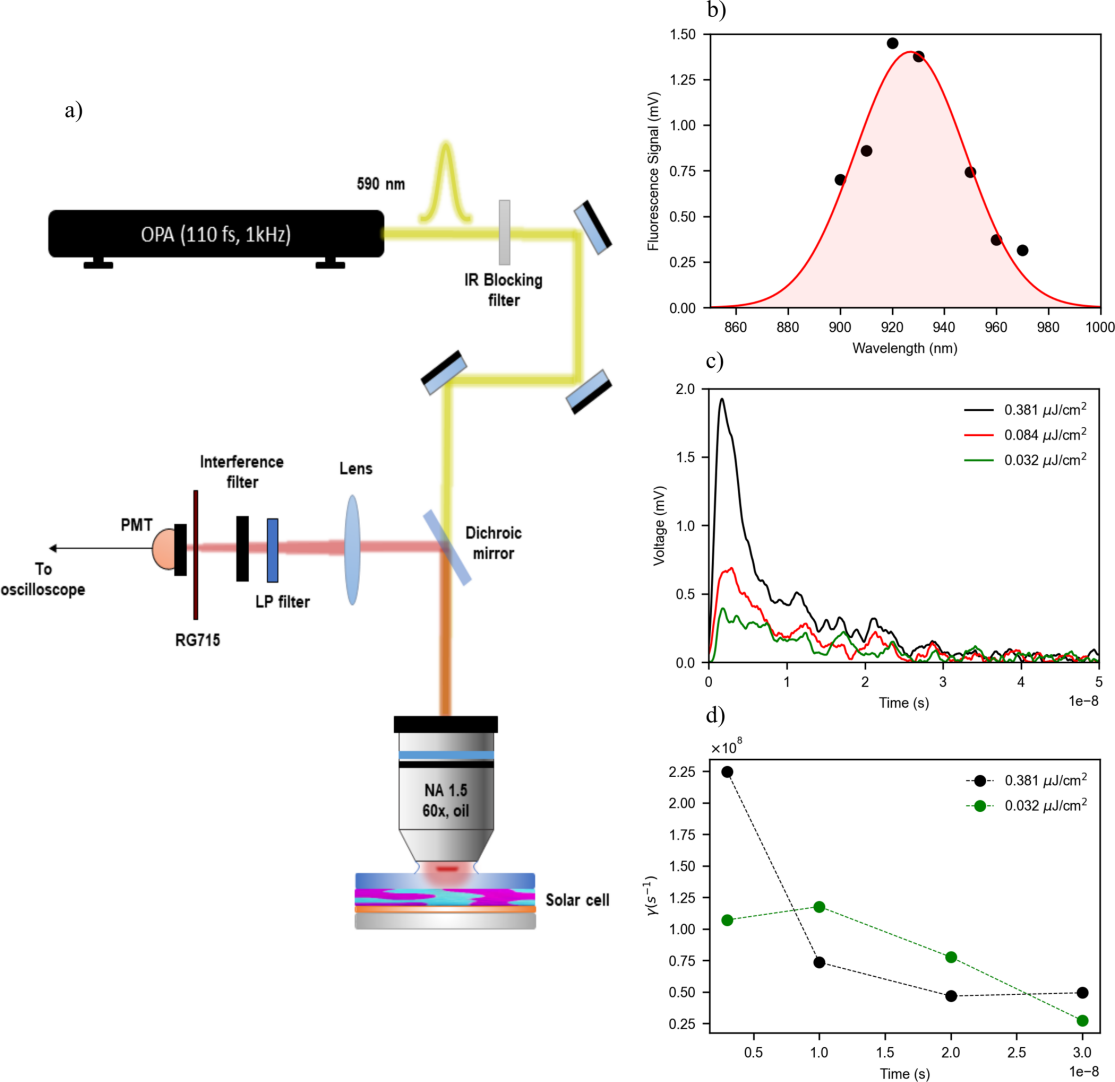
Time-dependent fluorescence. (a) Schematic illustration of the experimental setup used to measure the fluorescence in open-circuit of completed cells illuminated with a 1 kHz train of 120 fs laser pulses tuned at 590 nm. (b) External cell fluorescence when the pump energy density per pulse was 0.38 μJ/cm^2^. The fluorescence signal was filtered using several interference filters centered at 900, 910, 920, 930, 950, 960, and 970 nm. The experimental data points are corrected by the corresponding blend absorptions at each wavelength. The red line corresponds to a Gaussian fit of the corrected experimental data points. (c) Fluorescence time evolution when pumping at three different pulse energy densities. (d) Time evolution of the charge density decay rate extracted from the normalized time derivative of the fluorescence measurements from (c) at 0.38 μJ/cm^2^ (black solid dots) and 0.032 μJ/cm^2^ (green solid dots) under the assumption that the charge density is proportional to the fluorescence measured by the PMT.

A nongeminate character of the recombination at high pump intensities or short delay times is confirmed by a fluorescence decay time strongly dependent on the photon pump fluence, as seen in Figure 4c. Indeed, when the system brought many *k*_B_ *T* out of equilibrium, the excess electron and hole charge densities are much larger than their corresponding equilibrium densities, and direct recombination becomes faster than the trap-assisted one. As time elapses or when the pump photon fluence is reduced, charge density (*n*) in the bands would be reduced, too. Then, as seen in Figure 4d, the charge density decay rate (γ = (1/*n*)­d*n*/d*t*) converges to ∼4 × 10^7^ s^–1^, which we may assume as being close to the trap-assisted recombination rate. At large delays relative to the excitation pulse, when the charge population at the bands is largely depleted, such a later mechanism would dominate over the radiative one. Such a strong time dependence of the charge density decay rate confirms that direct recombination plays a relevant role in the *V*_oc_ dynamics.

## 4 Conclusions

We conclude that in completed organic solar cell devices based on the PM6:Y6 blend, the fluorescence originating by the blend/charge transporting layer interfaces from nongeminate radiative recombination is one of the mechanisms governing the *V*_oc_ dynamics. Even though the overall FQY is less than 1%, relatively small changes in such an FQY already have a measurable impact on the QFLS or *V*_oc_. We demonstrated that an 18% increase in the FQY, reached after the addition of a LiF passivation layer on the ETL, corresponds to a significant reduction in trap-assisted recombination, leading to an increase in *V*_oc_ as high as 12 mV, in very good agreement with the estimate from the two-diode model used to model the performance of the PM6:Y6 organic solar cell. Such interfacial-based *V*_oc_ increase could be combined with bulk-based approaches, such as molecular engineering (20–30 mV),^5,31^ electron–phonon suppression (30–40 mV),^33^ and delocalization of exciton wave function (10–70 mV),^10^ to obtain very significant *V*_oc_ gains in OSCs.

It is worth remarking that the type of fluorescence seen is not specific to the blend used. Provided it is observed in completed cells when the light-absorbing layer/charge transporting layer interfaces are present, it would be worth investigating if in other kinds of planar junction solar cells recombination resulting from fluorescence near such interfaces may also play a relevant role in governing the *V*_oc_ dynamics. Finally, the work we report opens the door to further limit *V*_oc_ losses by restricting the solid angle for the emission of photons, a *V*_oc_ loss reduction which has been numerically determined to be almost 300 mV.^13^

## Supplementary Material



## Notes

Interface bands analysis; steady-state fluorescence measurements; fluorescence quantum yield determination; radiative recombination current density; FQY and *V*_oc_ versus light intensity in the two-diode equivalent circuit model; and impact of LiF on series resistance and trap recombination current density (PDF)
