# Supplementary material for: Nongeminate Radiative Recombination and V oc in Organic Solar Cells Enhanced by a Charge Transporter/Absorber Interface Change
Source: ACS Appl Energy Mater. 2025 Dec 3;8(24):17863–70. doi: 10.1021/acsaem.5c02775 (PMC12728799; doi:10.1021/acsaem.5c02775)
Supplement: Supplementary file 1 [file ae5c02775_si_001.pdf]

Supporting Information

Nongeminate Radiative Recombination and Voc in Organic Solar Cells Enhanced by a Charge Transporter/Absorber Interface Change

Francisco Bernal-Texca[†*], *Chiara Cortese*[†], *Mariia Kramarenko*[†], *and Jordi Martorell*[†,ǂ,*]

[†]ICFO-Institut de Ciencies Fotoniques, The Barcelona Institute of Science and Technology, 08860 Castelldefels, Barcelona Spain.

[ǂ] Departament de Física Universitat Politécncnica de Catalunya, Terrasa 08222, Spain.

[*]Email: francisco.bernal@icfo.eu

[*]Email: jordi.martorell@icfo.eu

Contents:

Section S.1: Interface bands analysis S2

Section S.2: Steady-state fluorescence measurements S2

Section S.3: Fluorescence quantum yield determination S2

Section S.4: Radiative recombination current density (J_r) S4

Section S.5: V_{oc} versus light intensity and FQY in the two-diode equivalent circuit model S6

Section S.6: Impact of LiF on series resistance and trap recombination current density from dark J-V curves and FQY S7

Supplementary figures S9

Supplementary tables S22

References S23

Section S.1: Interface bands analysis

The UPS spectra in **Figure 2c** show a decrease in the work function (W_f) of the ZnO with the addition of the LiF, from 3.62 eV to 3.12 eV. This reduction explicitly confirms the passivation effect of the LiF layer on the ZnO surface. The bandgap of these materials was independently determined from Tauc plots derived from absorption data (**Figure S10**). Based on these experimental findings, the schematic description of the interface band bending for the ETLs and the Y6 electron acceptor after contact is illustrated in **Figure S11**. Initially, the ZnO's electron affinity (3.07 eV) is lower than that of the Y6 (4.26 eV). Upon contact, this difference causes the ZnO bands to bend upwards and the Y6 conduction band to bend downwards, creating an energy offset of 1.18 eV. The addition of the LiF reduces this energy offset significantly. With LiF in place, the bands become nearly flat at the interface, resulting in a minimised energy offset of only 0.71 eV. This optimisation in band alignment is the mechanism that facilitates more efficient charge extraction with the addition of the LiF.

Section S.2: Steady-state fluorescence measurements.

For the steady state fluorescence measurements of the different samples, we illuminated them with 20 mW of visible light at 532 nm from a CW Nd:YAG Coherent Compass laser. The laser beam was focused onto the different samples with a beam diameter of 0.5 cm. To detect the emitted light from the different samples, we used a photomultiplier tube (PMT) Hamamatsu R5108 which aperture was placed at a distance (*d*) of 70 mm and at 30º with respect to the sample surface normal as shown in the schematic representation of the setup displayed in **Figure S13**. To obtain the fluorescence spectra in wavelength steps of 10 nm, we used a total of nineteen bandpass filters with peaked transmission wavelength ranging from 810 to 990 nm (FBH series from Thorlabs). The FWHM of all the bandpass filters used was 10 nm. Additionally, a long pass RG715 filter was also placed in between the bandpass filter and the PMT to eliminate any residual signal coming from the pump beam. To obtain an accurate quantitative estimate of the fluorescence quantum yield (FQY), the 7 mm diameter (*D*) circular aperture was placed in front of the PMT and filters to ensure that the fluorescence light illuminated only the homogeneous photosensitivity part from the PMT photocathode.

Section S.3: FQY determination

To determine the FQY of completed solar cells we may assume that upon illumination by the laser light, charge recombination photons are emitted in all directions equally. Then, the specific intensity in the forward or backward directions $I^{\pm}$ within the active layer of the cell, corresponding to the power emitted by a surface area *da* in a solid angle $d\Omega$ and within a frequency range between ν and $\nu + d\nu$, would be isotropic, as schematically shown in **Figure S14**.

From an optical perspective the completed solar cell can be considered as a light absorbing thin layer sandwiched between a highly reflective mirror (HRM) with reflectivity power intensity *R* and a *partially transmissive layer* with light transmission power intensity T_{ia} and a reflectivity power intensity R_{ia}. When considering infinite multiple reflections on both sides of the interface limits of such absorbing layer, it is straightforward to show that the internal intensity from the fluorescent photons incident on the front interface can be expressed as

$$I_i = I^{-} \frac{1}{1-x} \qquad \text{(Eq. S1)}$$

Where

$$x = R_{ia} R e^{-2\frac{4\pi}{\lambda_e} k \frac{l}{\cos\theta_i}} \qquad \text{(Eq. S2)}$$

being l the thickness of the light absorbing layer, k the imaginary part of the refractive index at the wavelength of photon emission, λ_e the wavelength for the emitted photons in vacuum, and θ_i the angle of incidence for such emitted photons onto the partially transmissive layer from inside as shown in **Figure S14**.

Assuming that the circular aperture with diameter *D* in front of the PMT is placed at a large distance *d* from the front cell interface as shown in **Figure S13**, one can express the power measured by the PMT in terms of the emitted photon transmitted intensity I_t through an area *da* as

$$dP_m = I_t cos\theta_a d\Omega_a dadv \quad \text{(Eq. S3)}$$

where θ_a is the angle subtended by the axis passing through the aperture and cell centers and the surface normal, while $d\Omega_a$ is the solid angle subtended by the aperture viewed from such cell center. By relating the transmitted intensity to the incident intensity, one can express, as schematically shown in **Figure S15**, such measured power in terms of the power incident from inside the cell onto the same interface surface area *da* as

$$dP_m = T_{ia} I_i cos\theta_i d\Omega_i dadv \quad \text{(Eq. S4)}$$

By using Snell's law and Eq. (S1) relating the incident intensity to the specific intensity, the measured power by the PMT can be written in terms of the solid angle in the outside air medium ($d\Omega_a$) as

$$dP_m = T_{ia} daI^{-} \frac{1}{1-x} \frac{n_a^2 cos\theta_a}{n_i^2 cos\theta_i} d\Omega_a dv \quad \text{(Eq. S5)}$$

Where n_a and n_i are the refractive indexes of air and the active layer, respectively. By integrating over the 4π solid angle one can obtain the emitted power by a small area *da* in a frequency range between ν and $\nu + d\nu$ in terms of the power measured by the PMT

$$dP_e = 2\pi dadvI^{-} = \frac{2\pi dP_m(1-x)}{T_{ia} d\Omega_a} \frac{n_i^2 cos\theta_i}{n_a^2 cos\theta_a} \quad \text{(Eq. S6)}$$

If we assume a gaussian profile for the interference filter used in front of the PMT and when the emitted light is assumed to also have a gaussian profile, the total full bandwidth emitted power dP_{et} can be shown to be

$$dP_{et} = \frac{FWHM_e}{FWHM_f} dP_e \quad \text{(Eq. S7)}$$

where $FWHM_e$ and $FWHM_f$ correspond to the full width half maximum of the total emitted power and interference filter, respectively. From there we may readily obtain the energy fluorescence quantum

yield (FQY_e) dividing dP_{et} by the incident laser light power P_l corrected by the external quantum efficiency (EQE) at the laser wavelength.

$$FQY_e = \frac{dP_{et}}{EQE(\lambda_l)P_l A_c} \quad \text{(Eq. S8)}$$

Where A_c is the cell area which, if small compared to the distance d from the aperture in front of the PMT to such cell, we may assume to be equal to the small area da for the photon emission found in dP_{et}. To obtain the photon number FQY, FQY_e must be corrected by the ratio between the photon emission wavelength and laser wavelength

$$FQY = FQY_e \frac{\lambda_e}{\lambda_l} \quad \text{(Eq. S9)}$$

For the completed cells on glass substrates with a front ITO layer and an Ag back contact, the T_{ia} and R_{ia} are shown in **Figure S16** as a function of the light angle of incidence to the blend/ITO interface (θ_i). Assuming that the PMT is placed a $\theta_a = 30°$ with respect to the normal to the cell, then $\theta_i = 14.5°$. Taking the corresponding values for such T_{ia} and R_{ia} at $\theta_i = 14.5°$, $FWHM_f = 10\ nm$, $\lambda_e = 920\ nm$, $\lambda_l = 532\ nm$, $EQE(\lambda_l) = 0.85$, d = 70 mm, and D = 7 mm, we can estimate the ***FQY* to be 0.52%** for the reference ZnO cell where $FWHM_e = 87\ nm$, and 0.60% for ZnO/LiF cell where $FWHM_e = 104\ nm$.

Section S.4: Radiative recombination current density (J_r)

The radiative recombination current density in a planar geometry solar cell at a temperature T_c can be associated to the absorption of thermal photons in the region (i) where photon emission takes place. The number of such thermal photons within a solid angle $d\Omega_a$ at a given frequency in a frequency range between ν and $\nu + d\nu$ that are incident on an area da at the interface that separates the cell from air (See **Figure S17**) is given by

$$dn = cos\theta_a d\Omega_a da \frac{2}{c^2} \frac{\nu^2 d\nu}{e^{h\nu/k_B T_c} - 1} \quad \text{(Eq. S10)}$$

We can define an effective thermal photon flux as

$$\phi_r = \frac{dn}{d\Omega_a da d\nu} \quad \text{(Eq. S11)}$$

Then, the radiative recombination current density associated to such thermal photon absorption in a volume $dV = dadz$ would be given by

$$dJ_r = q dz d\Omega_a \int_0^\infty Abs_i(z,\theta,\nu)\phi_r(\nu)d\nu \quad \text{(Eq. S12)}$$

where q is the electron charge and $Abs_i(z,\theta,\nu)$ is the photon absorption probability in the region (i).

The time averaged loss of electromagnetic energy per unit time in a volume $dV = dadz$ is given by

$$du_{EM} = -\frac{1}{2} Re\{\mathbf{E} \cdot \mathbf{J}_d^*\} dV \quad \text{(Eq. S13)}$$

Under the assumption of harmonic waves, the displacement current density $\mathbf{J}_d$ can be written as

$$\mathbf{J}_d = \frac{\partial \mathbf{D}}{\partial t} = -i\omega\epsilon\mathbf{E} = -i\omega(\epsilon' + i\epsilon'')\mathbf{E} \quad \text{(Eq. S14)}$$

Since the imaginary part of the dielectric function in layer (*i*) is

$$\epsilon_i^{''} = 2n_i k_i \varepsilon_0, \quad \text{(Eq. S15)}$$

where n_i and k_i are the real and imaginary parts of the refractive index for layer (*i*), respectively. Then, the loss of electromagnetic energy can be rewritten as

$$du_{EM} = -\frac{1}{2}\frac{2\pi c}{\lambda} 2n_i k_i \varepsilon_0 |\mathbf{E}_i|^2 dadz \quad \text{(Eq. S16)}$$

where λ is the wavelength of the electromagnetic wave in vacuum. Under the assumption that the time averaged incident intensity is

$$I_o = -\frac{1}{2} n_a c \varepsilon_0 |\mathbf{E}_a|^2, \quad \text{(Eq. S17)}$$

we may obtain the fraction of electromagnetic energy absorbed or the probability for photon absorption in the layer (*i*) as:

$$Abs_i(z,\theta,\nu) = \frac{4\pi c}{\lambda}\frac{n_i(\nu)}{n_a} k_i(\nu) \frac{|\mathbf{E}_i(z,\theta,\nu)|^2}{|\mathbf{E}_a|^2} \quad \text{(Eq. S18)}$$

where $k_i(\nu)$ is modelled from the PM6:Y6 absorption coefficient measured experimentally incorporating an exponential decay corresponding to the Urbach tail which accounts for localized states below the band edges[1] (see **Figure S18**)

Under the assumption that such region (*i*) is homogenous, one may write an electromagnetic field satisfying Maxwell's equations as the sum of a forward and backward propagating plane waves

$$\mathbf{E}(z,\theta,\nu) = \mathbf{E}_{ip}^{+}(\theta,\nu)e^{+i(\mathbf{k}_i\cdot\mathbf{r}-\omega t)} + \mathbf{E}_{ip}^{-}(z,\theta,\nu)e^{-i(\mathbf{k}_i\cdot\mathbf{r}-\omega t)} \quad \text{(Eq. S19)}$$

being p either the TE or TM component of the electric field, $\mathbf{E}_{ip}^{\pm}(\theta,\nu)$ and $\mathbf{k}_i$ the constant field amplitudes and complex wave vector in the layer (*i*), respectively. Provided the region (*i*) is part of a cell with a layered architecture, which dimensions in a direction perpendicular to *z* (cf. **Figure S17**) are assumed to be infinite, the only nonvanishing component of the imaginary part of $\mathbf{k}_i$ would be in the *z* direction, while its real part would be perpendicular to the planes of constant phase. Under such layered configuration for the solar cell, one may use the transfer matrix formalism to determine the $\mathbf{E}_{ip}^{\pm}(\theta,\nu)$ constant amplitudes in terms of the amplitude of the incident field, and then, readily, obtain the fraction of energy absorbed in the layer (*i*), or the probability for thermal photon absorption. From there, using Eq. (S12) we can compute the radiative recombination current as

$$J_r = q\int dz \int_{2\pi} d\Omega \int_0^{\infty} \eta(Abs_{iTE}(z,\theta,\nu) + Abs_{iTM}(z,\theta,\nu))cos\theta \frac{1}{c^2}\frac{\nu^2 d\nu}{e^{h\nu/k_B T_c} - 1} \quad \text{(Eq. S20)}$$

where η is the internal quantum efficiency.

Using Eq. (S20), the experimental refractive indices reported in Section S9, $\eta = 0.92$ and integrating in the range (expressed in wavelength) 300 nm-1800 nm, J_r at 0 V was determined to be $1.725 \cdot 10^{-20}$ A/cm^2. The introduction of 5 nm of LiF in the optical model provides an increase in J_r at 0 V of $0.018 \cdot 10^{-20}$ A/cm^2, amounting to 1% of the reference cell J_r.

Section S.5: Open circuit voltage (V_{oc}) versus light intensity and FQY in the two-diode equivalent circuit model

Under steady-state illumination, the dynamics of electron and hole distribution in a single junction semiconductor solar cell is regulated by the splitting of the quasi-Fermi levels (QFLs), which in open circuit conditions is maintained by the balance of the electron-hole creation and recombination current densities.
It has been shown that in open circuit conditions, the voltage versus illumination intensity can be reasonably well described by the equivalent circuit of the two-diode model shown in **Figure S19**, where the electron-hole pumping is achieved by the short circuit current (J_{sc}) and its deactivation is carried out by an electric leakage from direct recombination (J_d), a recombination due to trap states (J_t), a finite shunt resistance (R_{sh}) and a series resistance (R_s). As discussed in the main text, J_d can be separated into a radiative and a nonradiative contributions as:

$$J_d = J_r + J_{nr} \qquad \text{(Eq. S21)}$$

In principle J_t is also the sum of a radiative ($J_{t,r}$) and nonradiative ($J_{t,nr}$) contribution. However, given the higher ideality factor $n = 2$ associated to trap-mediated recombination, and evidences in other works[6] for the ratio between $J_{t,r}$ and $J_{t,nr}$ to be $\sim 10^{-8}$, we neglected any trap-mediated radiative contribution.

According to the two-diode model, the J-V curve is described by:

$$\left[J_d\left(e^{\frac{q(V-JR_sA_c+V_{off})}{k_BT}}-1\right)+J_t\left(e^{\frac{q(V-JR_sA_c+V_{off})}{2k_BT}}-1\right)+\frac{V+V_{off}}{A_cR_{sh}}-J_{sc}\right]\frac{R_{sh}}{R_s+R_{sh}}-J=0 \qquad \text{(Eq. S22)}$$

where q is the electric charge, k_B the Boltzmann constant, T the cell temperature, A_c the cell area, and V_{off} is a voltage offset accounting for the difference between the QFLs splitting (QFLS) internal to the cell and the externally probed voltage[4]. For organic solar cells this offset has been related to the QFLs pinning at the interfaces with the ETL and HTL, and in the specific case of PM6:Y6, V_{off} has been measured experimentally to be in the order of several tenths of mV[2,3].

The QFLS energy can be correlated to the FQY through[4]:

$$QFLS = QFLS_{rad} + k_BT\ln(FQY) \qquad \text{(Eq. S23)}$$

Where $QFLS_{rad}$ is the radiative limit of the quasi-Fermi levels splitting, obtained as:

$$QFLS_{rad} = k_BT\ln\left(\frac{J_{sc}}{J_r}+1\right) \qquad \text{(Eq. S24)}$$

Finally, the total V_{oc} associated energy loss ($\Delta E_{nr,loss}$) due to nonradiative recombination processes can be estimated as $\Delta E_{nr,loss} = -qV_{off} + k_BT\ln(FQY)$.

In the two-diode equivalent circuit to model the solar cell, FQY is given by:

$$FQY = \frac{J_r(V)}{J_d(V)+J_t(V)+J_{sh}(V)} \qquad \text{(Eq. S25)}$$

Where:

$$J_{sh}(V) = \frac{V}{A_cR_{sh}} \qquad \text{(Eq. S26)}$$

$$J_d(V) = J_d\left(e^{qV/k_BT}-1\right) \qquad \text{(Eq. S27)}$$

$$J_r(V) = J_r\left(e^{qV/k_BT} - 1\right) \quad \text{(Eq. S28)}$$

$$J_t(V) = J_t\left(e^{qV/2k_BT} - 1\right) \quad \text{(Eq. S29)}$$

Note that since FQY depends on the magnitude of QFLS, V_{off} is to be set to zero in the FQY-related calculations.

Finally, by setting $J = 0$ in Eq. (S22), we obtain a transcendental equation that relates the V_{oc} to illumination intensity:

$$\left[J_d\left(e^{q(V_{oc}+V_{off})/k_BT} - 1\right) + J_t\left(e^{q(V_{oc}+V_{off})/2k_BT} - 1\right) + \frac{(V_{oc}+V_{off})}{A_cR_{sh}} - J_{sc}\right] = 0 \quad \text{(Eq. S30)}$$

In order to determine V_{off}, transfer matrix method was first used to determine J_{sc} under 1 sun illumination as:

$$J_{sc} = q \int dz \int_{\nu_{min}}^{\nu_{max}} \eta Abs_i(z, \nu;\ \theta = 0)\Phi_{sun}(\nu)d\nu \quad \text{(Eq. S31)}$$

where Φ_{sun} is the spectral flux density of the sun. Using the experimental refractive indices reported in Section S9, $\eta = 0.92$, the J_r values determined in the previous section, the experimental FQY values and Eq. (S18), (S31), (S23) and (S24), J_{sc}, $QFLS_{rad}$ and $QFLS$ were calculated for the ZnO and the ZnO/LiF case and shown in **Table S2**.

V_{off} was then obtained in the case of ZnO as $\frac{QFLS}{q} - V_{oc}^{exp}$, where V_{oc}^{exp} is the experimental average V_{oc} reported in Table 1 of the main text, leading to $V_{off} = 104$ mV. Note that the $QFLS$ energy difference corresponds to a voltage increase of 4.0 mV in the ZnO/LiF case, very close to the minimum V_{oc} enhancement reported in the main text. Therefore, $V_{off} = 104$ mV was kept constant in all the following calculations, and lead to $\Delta E_{nr,loss} = 0.240$ eV in the ZnO case, and $\Delta E_{nr,loss} = 0.2360$ eV in the ZnO/LiF case

Using Eq. (S30) and V_{off} to fit the experimental V_{oc} vs illumination intensity data shown in **Figure S20**, we estimated J_d and J_t at 0V, and R_{sh} for the ZnO cell. The results of the fit are reported in **Table S3**.

Using Eq. (S25) for the FQY, Eq. (S30) with V_{off} = 0 mV to solve for the $QFLS$ voltage at 1 sun, and with V_{off} = 104 mV to solve for the V_{oc}, J_t, R_{sh} from **Table S3**, and J_{sc} from **Table S2**, the J_{nr} matching the average experimentally measured $V_{oc} = 841$ mV and $FQY = 0.52\%$ at 1 sun was estimated to be $1.85 \cdot 10^{-18}$A/cm^2. The corresponding $J_d = 1.87 \cdot 10^{-18}$A/cm^2 calculated with Eq. (S21) exhibits good agreement with the value reported in **Table S3**.

Section S.6: Impact of LiF on series resistance and trap recombination current density from dark J-V curves and FQY

J-V curves were measured in dark conditions and fitted with Eq. (S22), $J_{sc} = 0$, for the ZnO and ZnO/LiF case to assess the effect of LiF on the series resistance and on the trap current density. **Figure S21** shows the statistics of the fit parameters of interest, while **Figure S22** the raw data and fitting for respectively a representative ZnO cell curve and a Zno/LiF cell one. **Figure S21b** highlights a consistent increase in the series resistance in the ZnO/LiF case. **Figure S21a** further strengthen the claim of LiF effectively reducing J_t contribution to V_{oc}.

The experimental evidence for a relation between V_{oc} and FQY gains when the LiF is added in between the ZnO layer and the PM6:Y6 blend layer can be linked to a suppression in the trap recombination

using the equivalent circuit two-diode model. J-V curves under illumination of a representative ZnO cell and two ZnO/LiF cells displaying an experimental enhancement $\Delta V_{oc} = 5$ mV, 9 mV were fitted with Eq. (S22), as shown in **Figure S24**. From the fit results, reported in **Table S4**, it emerges that the parameter most affected by the LiF is J_t, dropping by $\sim 50\%$ in the case of $\Delta V_{oc} =$ 9 mV compared to the reference ZnO cell. In addition, when the fit parameters are substituted in Eq. (S25) to predict the FQY of the devices, in the case of $\Delta V_{oc} =$ 9 mV the gain in FQY reaches 36%, compared to the 18% obtained for $\Delta V_{oc} = 5$ mV.

Furthermore, when applying Eq. (S25) and (S30), all the (J_{nr}, J_t) pairs compatible with the observed 18% increase in fluorescence, considering FQY=0.50% as reference, lead to a 4 mV increase in V_{oc}, shown in **Figure S23c**. **Figure S23a, b** highlights the FQY increase when (J_{nr}, J_t) pairs leading to higher ΔV_{oc} are considered.

Supplementary Figures

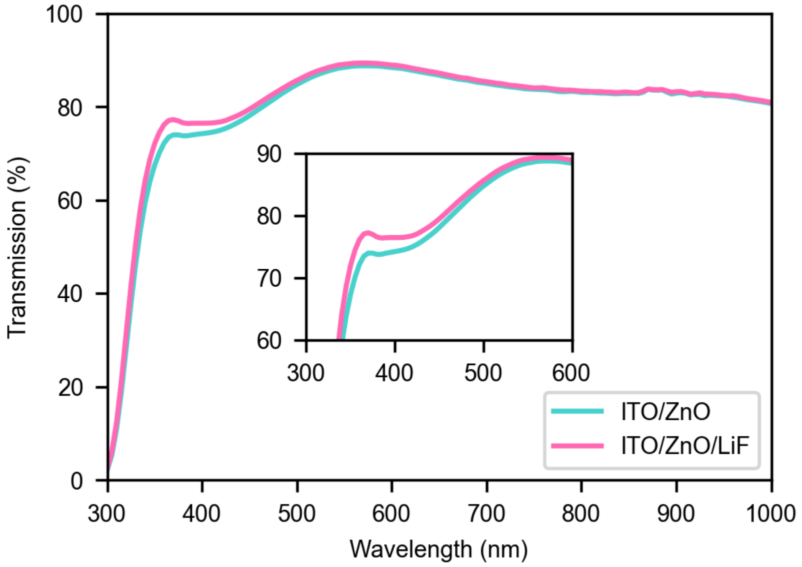

Figure S1. Transmission spectra of the ITO-coated glass substrates with ZnO and ZnO/LiF ETLs.

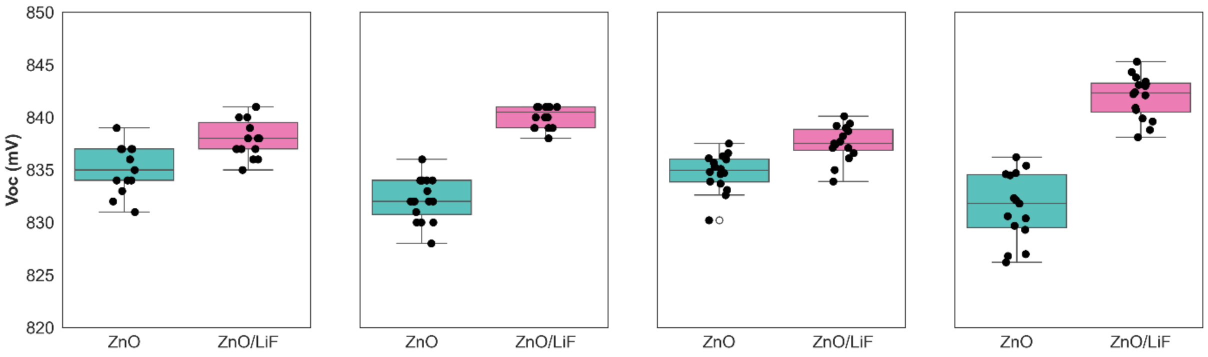

Figure S2. Four additional experiments using PM6:Y6 photoactive layer with the ETL being ZnO and ZnO/LiF. For all the cases, a V_{oc} enhancement is observed.

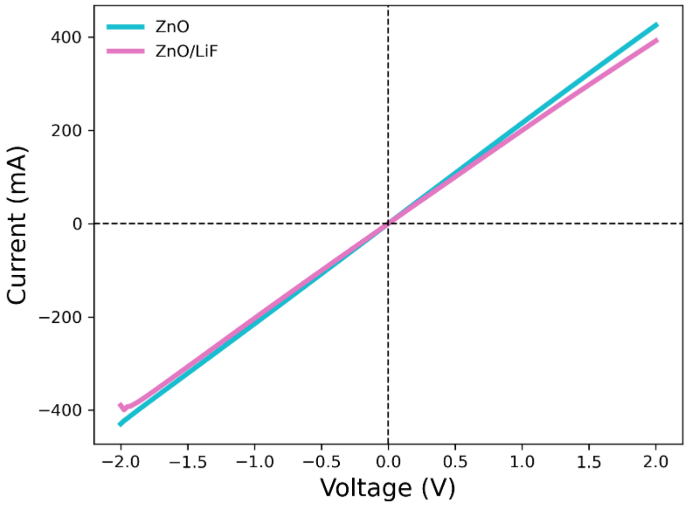

Figure S3. I-V measurement on the Substrate/ITO/ETL/Ag devices where the ETL is ZnO and ZnO/LiF.

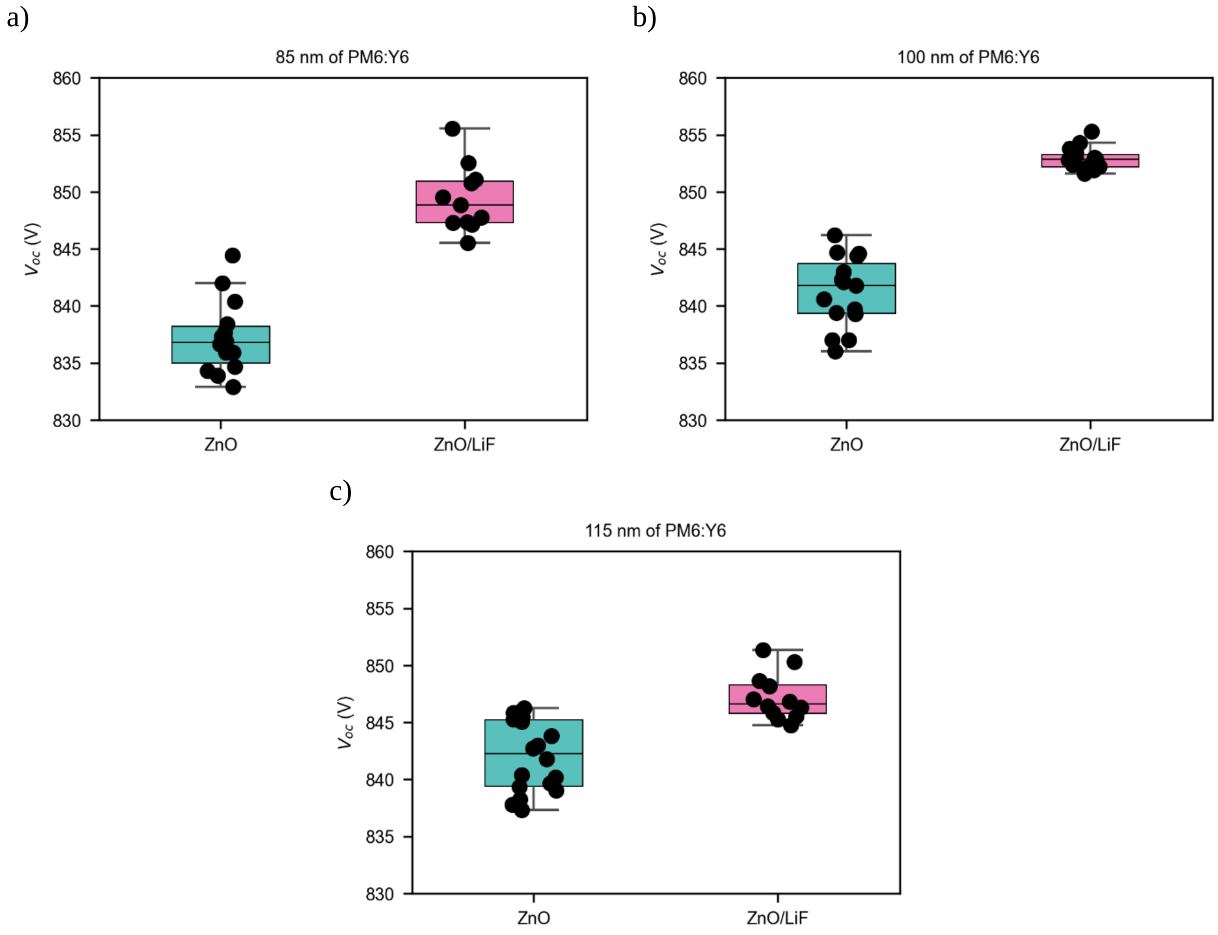

Figure S4. Experimental V_{oc} of the devices with a) 85 nm, b) 100 nm and c) 115 nm of PM6:Y6 active layer thicknesses.

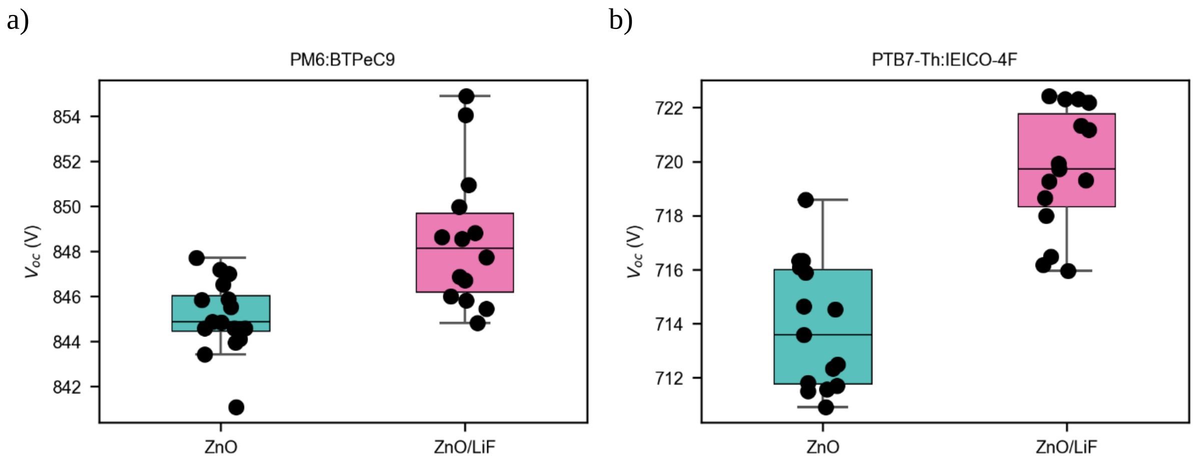

Figure S5. Experimental V_{oc} of the devices a) PM6:BTPeC9 and PTB7-Th:IEICO-4F with the two different ETLs: ZnO and ZnO/LiF.

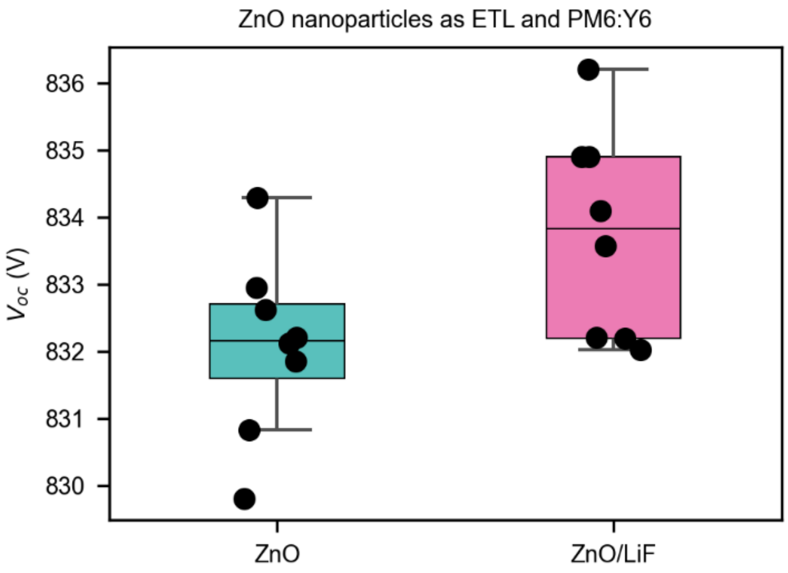

Figure S6. Experimental V_{oc} of the devices with commercially available ZnO nanoparticles (Avantama N-10-Jet) and ZnO nanoparticles with LiF deposited on top.

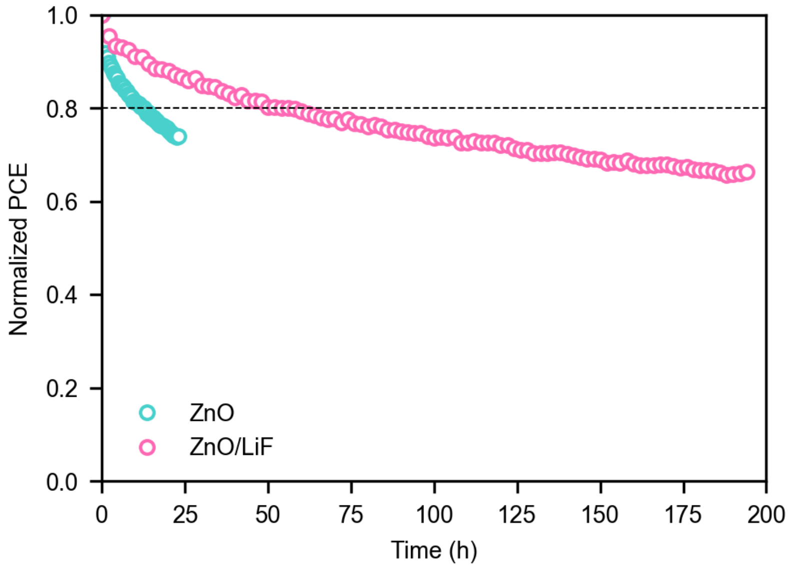

Figure S7. Stability test for the inverted devices with ZnO and ZnO/LiF as ETLs, under continuous light illumination using a white LED with light intensity equivalent to 1 sun in a N_2-filled glove box.

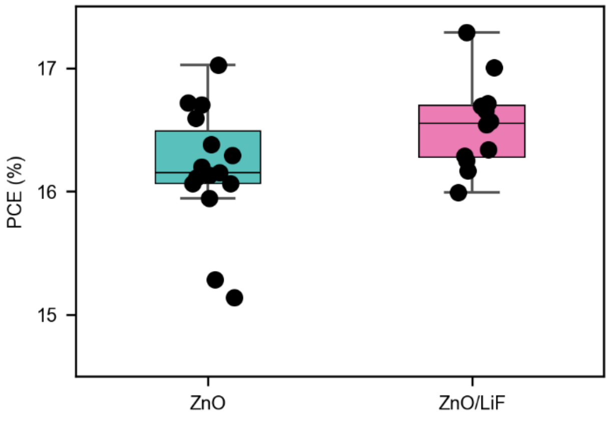

Figure S8. Statistics on the PCE from the complete cell measured under 1 sun AM15G illumination when ETL is a ZnO layer (left) and a ZnO layer with a LiF layer deposited on top (right).

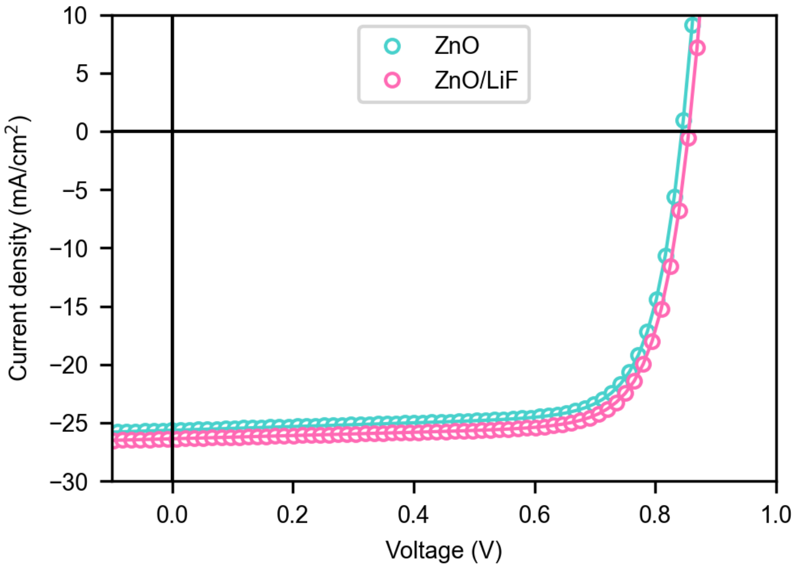

Figure S9. Experimental JV curves of the inverted devices with ZnO and ZnO/LiF ETLs.

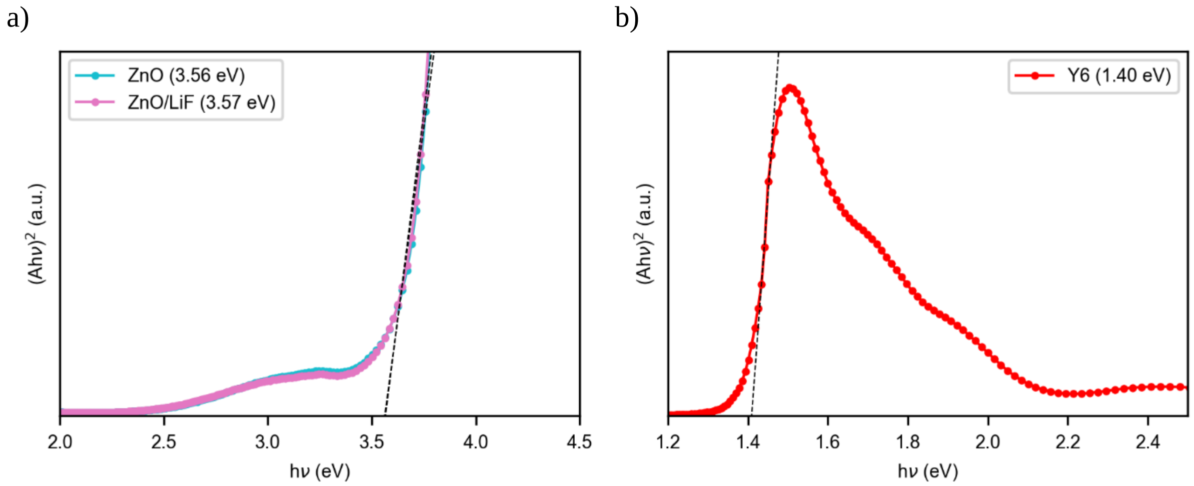

Figure S10. a) Tauc plot of ZnO and ZnO LiF from the absorbance spectra. b) Tauc plot of Y6 electron acceptor from its absorbance spectrum. The values in the legend correspond to the value of the optical bandgap.

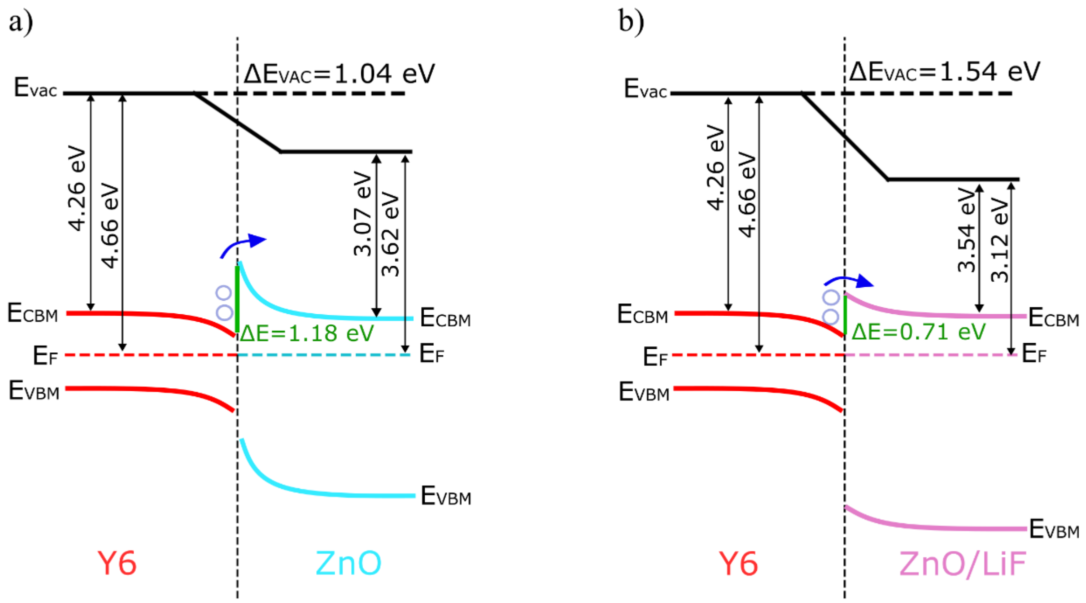

Figure S11. Schematic description of the interface band bending of a) ZnO and b) ZnO/LiF in contact with the electron acceptor Y6. The shift of vacuum energy level ($\Delta E_{Vac} = E_{Vac}^{Y6} - E_{Vac}^{ETL}$, where ETL corresponds to ZnO or ZnO/LiF) and the energy difference between the conduction band of ETL and Y6 ($\Delta E = E_{CB}^{Y6} - E_{CB}^{ETL}$) are shown in the figure. A summary of the experimental data of UPS and Tauc plots to reconstruct the band bending diagrams can be found in **Table S1**.

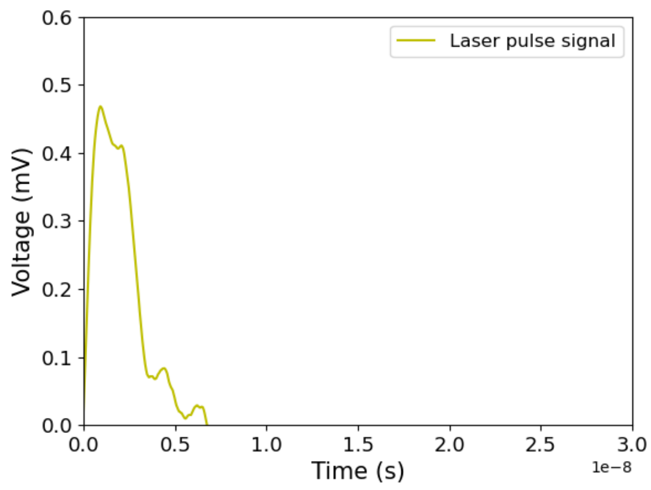

Figure S12. Laser pulse measured with the setup shown in **Figure 4a**.

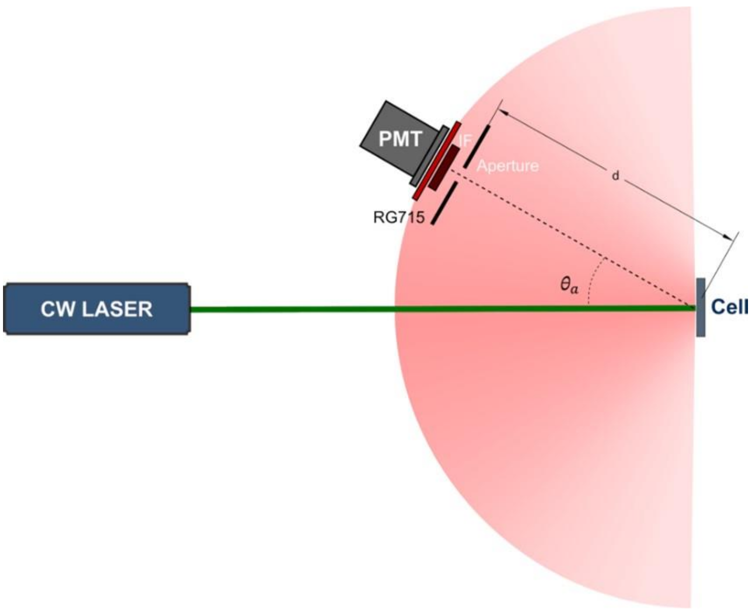

Figure S13. Setup for the steady-state fluorescence measurements

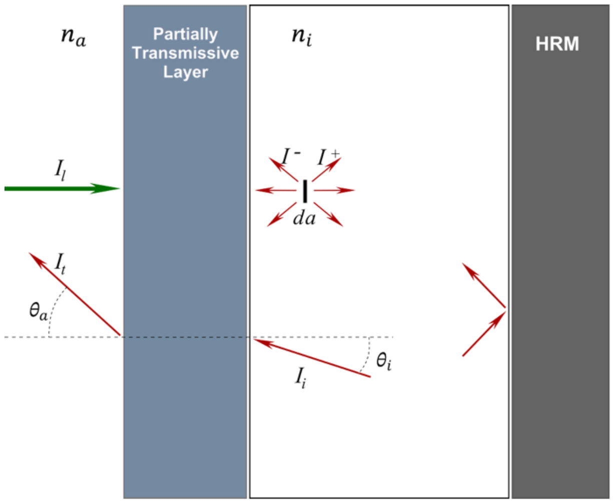

Figure S14. Schematic representation of the complete solar cell in the steady-state fluorescence measurements

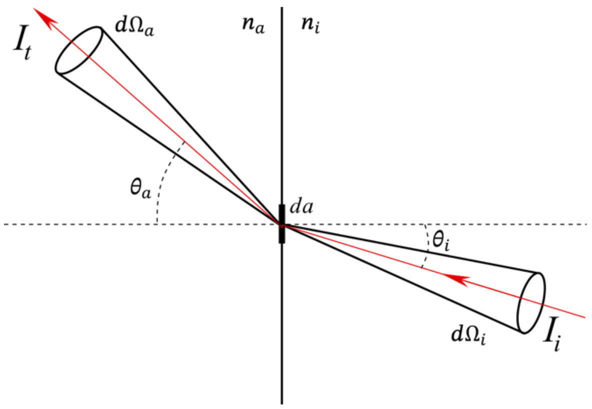

Figure S15. Solid angles at the air and active layer mediums

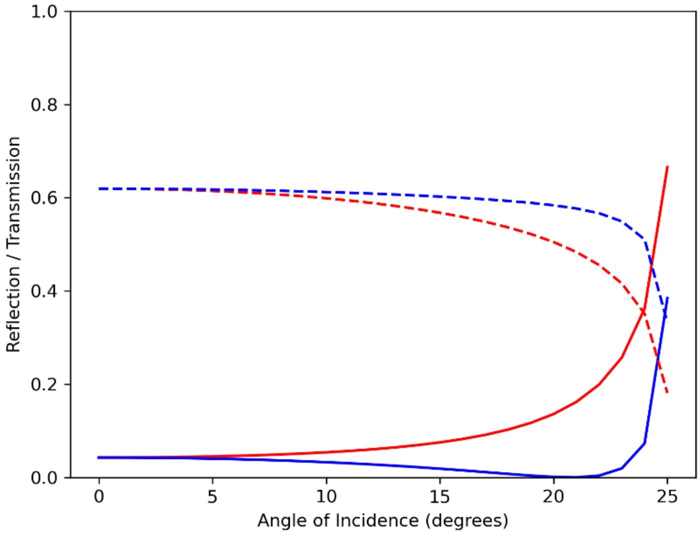

Figure S16. ZnO/ITO/Glass transmission (T_{ia}) (dashed) from the PM6:Y6 to air, and its reflection (R_{ia}) (solid) from PM6:Y6 back to PM6:Y6 as a function of θ_i at a wavelength of 910 nm.

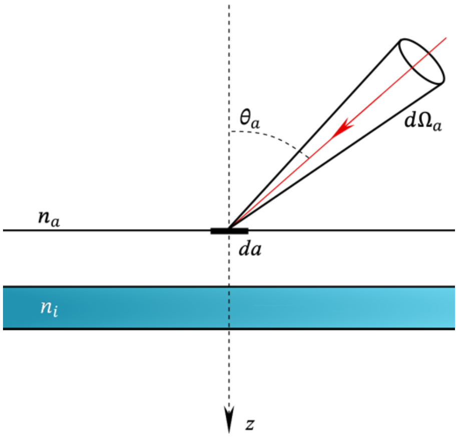

Figure S17. Thermal photon absorption at layer (i).

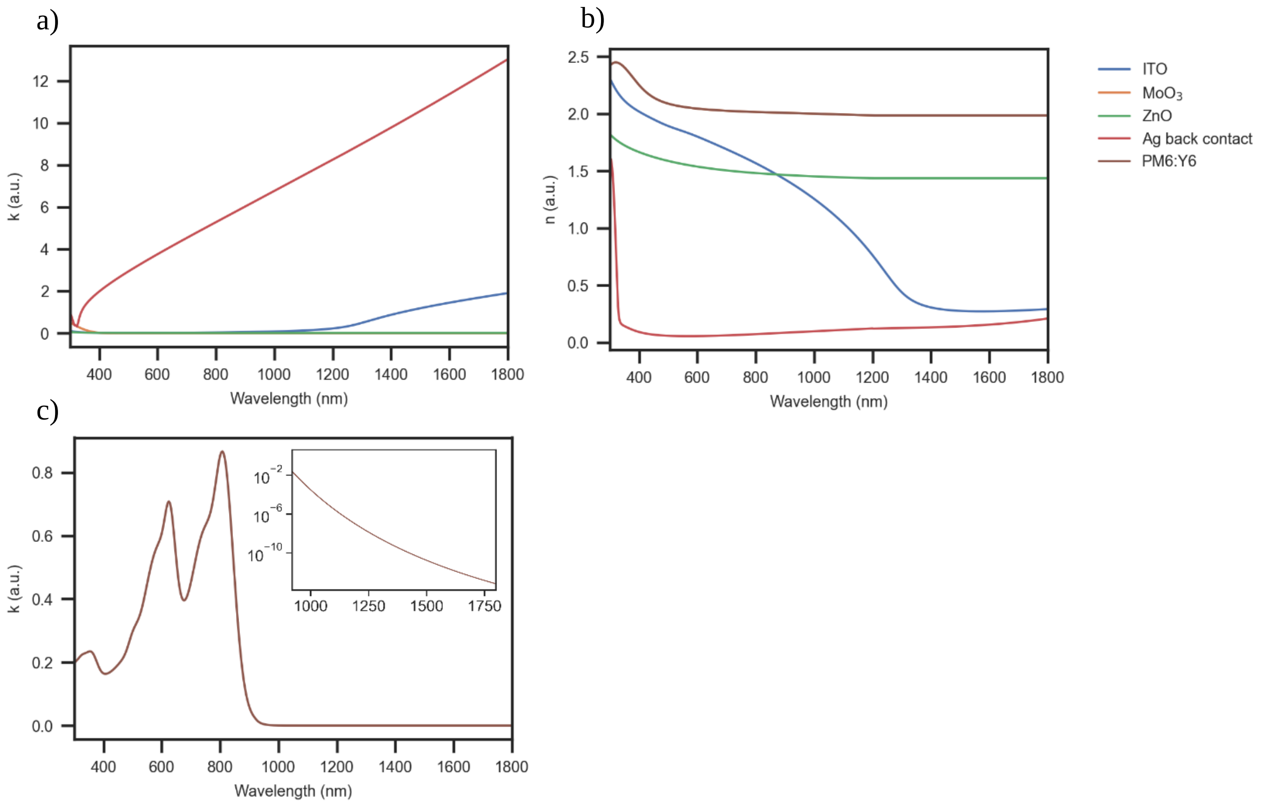

Figure S18. Wavelength dependency of real (a) and imaginary part (b) of the refractive indices used in the optical simulations. Panel (c) displays the imaginary part of PM6:Y6 refractive indeces, with the modeled Urbach tail part in the inset.

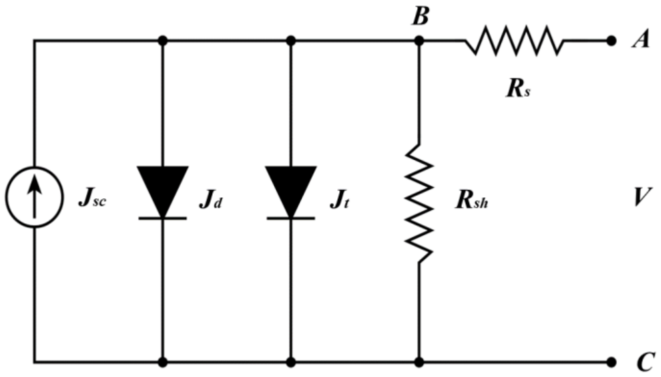

Figure S19. Equivalent circuit.

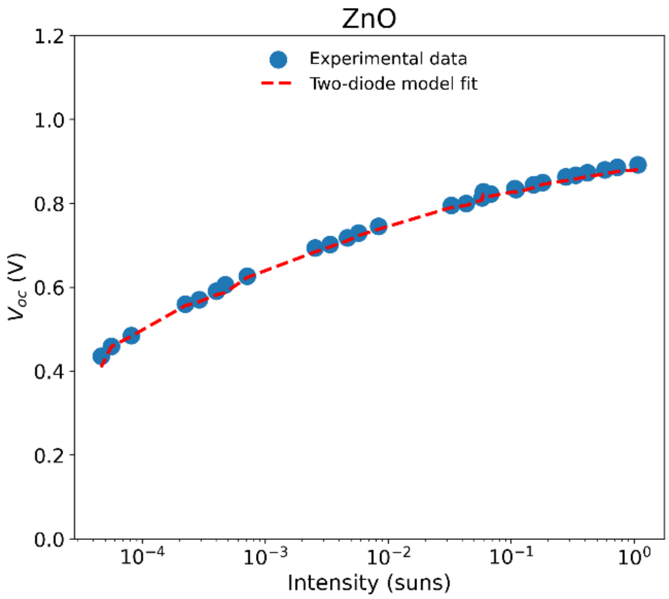

Figure S20. V_{oc} as a function of light intensity to determine J_d, J_t at 0V and R_{sh}.

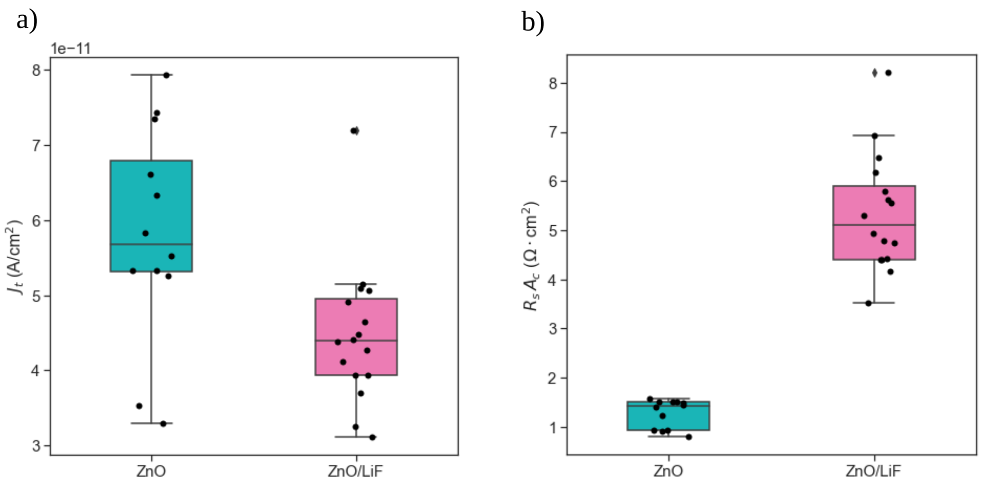

Figure S21. Statistics of (a) J_t at 0 V, and (b) R_sA_c, obtained from fitting 12 dark J-V curves of ZnO cells and 16 of ZnO/LiF cell with Eq. (S22), considering $J_{sc} = 0$ and $V_{off} = 104$ mV.

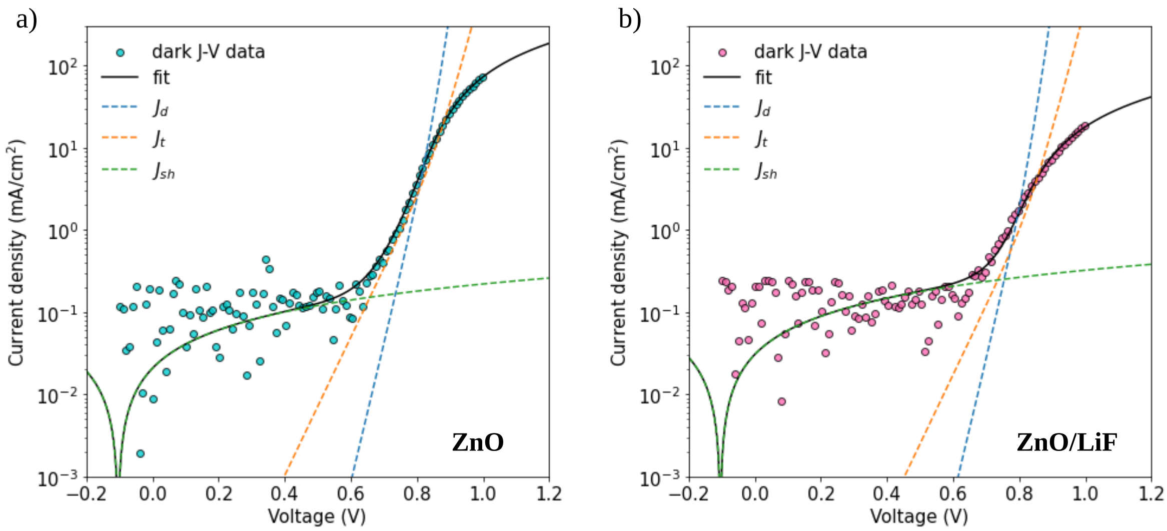

Figure S22. Dark J-V curves of representative (a) ZnO cell and (b) ZnO/LiF cell. To the raw data (circles) is superposed the fit (black line) with Eq. (S22), considering $J_{sc} = 0$ and $V_{off} = 104$ mV. The separate contributions from each parameter of the fit (dotted lines) were calculated using respectively Eq. (S29) for J_t (orange line), Eq. (S27) for J_d (blue line), and Eq. (S26) for J_{sh} (green line), with inclusion of V_{off}.

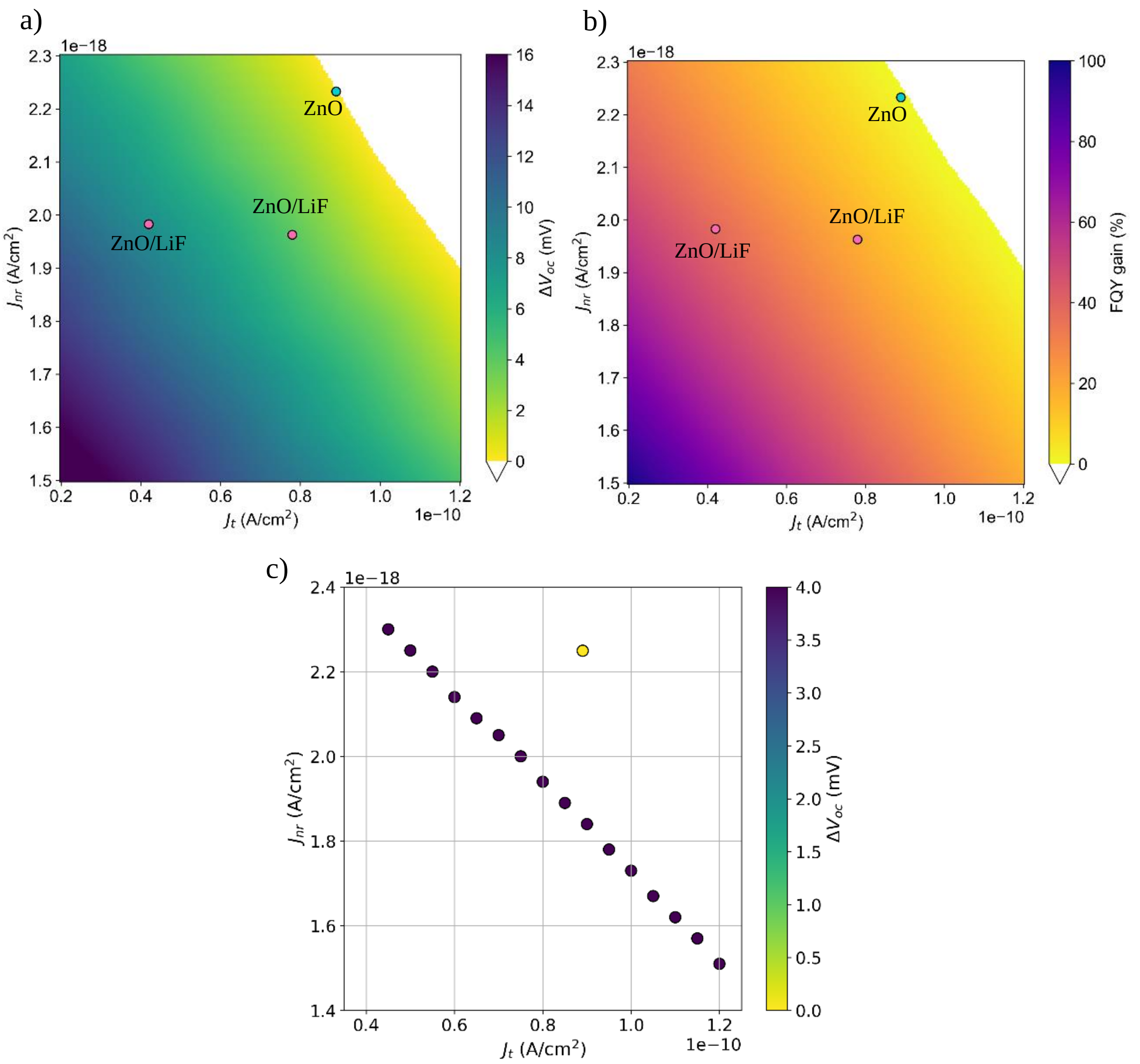

Figure S23. (J_{nr},J_t) pairs leading to (a) V_{oc} gain (ΔV_{oc}, off the paper axis), and the corresponding (b) FQY gain (off the paper axis, calculated with respect to reference cell: $\frac{FQY - 0.50\%}{0.50\%}$) predicted with the two-diode model. Superposed are the (J_{nr},J_t) pairs obtained from the fitting of experimental J-V curves of reference cell (blue dot), and two ZnO/LiF cells (pink dots) displaying respectively a 5 mV and 9 mV V_{oc} gain (see **Table S4**). (c) V_{oc} gain (ΔV_{oc}, off the paper axis) as a function of the (J_{nr},J_t) pairs (solid dots) obtained with the two-diode model and in agreement with the experimentally observed 18% FQY increase. The (J_{nr},J_t) pair for the reference cell, from **Table S4**, is also shown (yellow dot).

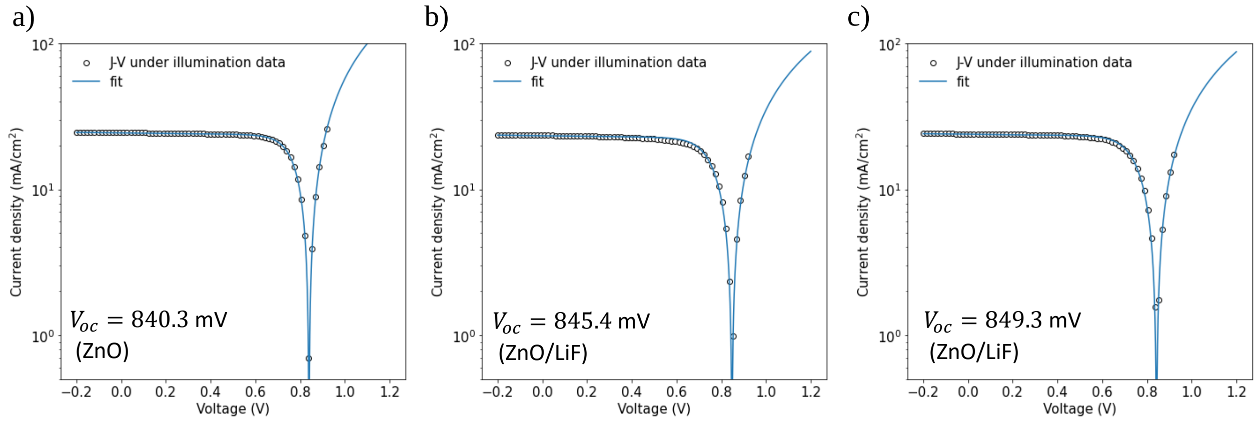

Figure S24. J-V data under illumination (dots) of (a) representative ZnO cell, (b) Zno/LiF cell with experimental 5 mV V_{oc} gain, and (c) Zno/LiF cell with experimental 9 mV V_{oc} gain. The experimental data were fitted with Eq. (S22) (blue line) to extract J_t, J_d, R_s, R_{sh} and V_{oc} given the simulated J_{sc}. Results are reported in **Table S4.**

Supplementary tables

Table S1. Summary of the data obtained from UPS and Tauc plots for ZnO, ZnO/LiF and Y6

Material	ϕ $[eV]$	E_{VB} $(vs\ E_F)$ $[eV]$	E_g^{opt} $[eV]$	IP $(IP = \phi + E_{VBM})$ $[eV]$	$E_{CB} = (IP - E_g^{opt})$ $[eV]$	$\Delta E = E_{CB}^{Y6} - E_{CB}^{ETL}$ $[eV]$
ZnO	3.62	3.01	3.56	6.63	3.07	1.18
ZnO/LiF	3.12	3.99	3.57	7.11	3.54	0.71
Y6	4.66*	1.00*	1.40	5.66	4.26	

*The UPS data of Y6 was obtained from Ref[5].

Table S2. Short circuit current density, quasi-Fermi level splitting in the radiative limit and considering the experimental FQY, voltage offset compared to V_{oc} and total nonradiative V_{oc} loss for ZnO and ZnO/LiF cell.

Cell type	FQY [%]	J_{sc} [mA/cm^2]	$QFLS_{rad}$ [meV]	$QFLS$ [meV]	qV_{off} [meV]	$\Delta E_{nr,loss}$ [eV]
ZnO	0.52	24.96	1081	945.1	104	-0.240
ZnO/LiF	0.60	24.99	1081	948.7	104	-0.236

Table S3. Current densities at 0V, shunt resistance and voltage offset for the ZnO cell.

Cell type	J_d $[\mathrm{A/cm^2}]$	J_t $[\mathrm{A/cm^2}]$	$R_{sh}A_c$ $[\Omega \cdot \mathrm{cm^2}]$	V_{off} $[\mathrm{mV}]$
ZnO	$2.0 \cdot 10^{-18}$	$0.65\ 10^{-10}$	$0.32\ 10^{6}$	104

Table S4. Comparison between experimental V_{oc} and calculated V_{oc} using two-diode model fitting from J-V curve under illumination for a ZnO cell and two ZnO/LiF cells displaying different V_{oc} gains. The fit parameters are reported together with the predicted FQY.

Cell type	Exp. V_{oc} [mV]	Fit V_{oc} [mV]	J_t [A/cm^2]	J_d [A/cm^2]	$R_{sh}A_c$ [kΩ·cm^2]	R_sA_c [Ω·cm^2]	FQY [%]
ZnO	840.3	840.2	$0.89 \cdot 10^{-10}$	$2.25 \cdot 10^{-18}$	1.00	1.4	0.50
ZnO/LiF	845.4	844.2	$0.78 \cdot 10^{-10}$	$1.98 \cdot 10^{-18}$	1.03	3.2	0.59
ZnO/LiF	849.3	848.2	$0.42 \cdot 10^{-10}$	$2.00 \cdot 10^{-18}$	1.01	3.8	0.68

References

1. van der Pol, T. P. A. *et al.* Origin, Nature, and Location of Defects in PM6:Y6 Organic Solar Cells. *Adv Energy Mater* **13**, (2023).

2. Riley, D. B. *et al.* Direct Quantification of Quasi-Fermi-Level Splitting in Organic Semiconductor Devices. *Phys Rev Appl* **15**, 1 (2021).

3. Phuong, L. Q. *et al.* Quantifying Quasi-Fermi Level Splitting and Open-Circuit Voltage Losses in Highly Efficient Nonfullerene Organic Solar Cells. *Solar RRL* **5**, 1–6 (2021).

4. Caprioglio, P. *et al.* On the Relation between the Open-Circuit Voltage and Quasi-Fermi Level Splitting in Efficient Perovskite Solar Cells. *Adv Energy Mater* **9**, (2019).

5. Karuthedath, S. *et al.* Intrinsic efficiency limits in low-bandgap non-fullerene acceptor organic solar cells. *Nat Mater* **20**, 378–384 (2021).

6. Zarrabi, N., *et al.* Charge-generating mid-gap trap states define the thermodynamic limit of organic photovoltaic devices. *Nat Commun* **11**, 5567 (2020).
